# CRISPR screening approaches in breast cancer research

**DOI:** 10.1007/s10555-025-10275-1

**Published:** 2025-07-12

**Authors:** Mark Samuels, Simoni Besta, Andrea Lauer Betrán, Reza Shirazi Nia, Xiaohong Xie, Xidong Gu, Qijin Shu, Georgios Giamas

**Affiliations:** 1 International Oncology Institute, The First Affiliated Hospital of Zhejiang Chinese Medical University, Oncology Department of the First Affiliated Hospital of Zhejiang Chinese Medical University, Hangzhou, 310053 China; 2 Department of Biochemistry and Biomedicine, School of Life Sciences, University of Sussex, JMS Building, Falmer, Brighton, BN1 9QG UK; 3 Department of Breast Surgery, The First Affiliated Hospital of Zhejiang Chinese Medical University, Hangzhou, 310053 China

**Keywords:** CRISPR, Screening, Drug discovery, Breast cancer, Functional genomics, Novel therapies, Drug resistance

## Abstract

The emergence of CRISPR-Cas9 technology has transformed functional genomics, offering unmatched opportunities to dissect and understand biological pathways and identify novel therapeutic targets in cancer. Breast cancer is a complex, heterogeneous disease and remains a major cause of morbidity and mortality in women, particularly when diagnosed at advanced or metastatic stages where effective treatments are limited. High-throughput CRISPR screening is undoubtedly a powerful tool to discover novel drug targets, uncover synthetic lethal interactions, and identify vulnerabilities in cancer. This review focuses on advances in our understanding of breast cancer developed through CRISPR-based screening technology, particularly in identifying drivers of breast cancer progression, growth, and metastasis, as well as in identifying potential new therapeutic targets and combination therapies. We discuss recent discoveries, current challenges, and limitations of this approach and explore how advancements in CRISPR technology could have a profound impact on the future of breast cancer treatment.

## Introduction

Breast cancer is the most common form of cancer and the largest cause of cancer death among women globally [1]. It is a heterogeneous disease with distinct molecular subtypes classified based on oestrogen receptor alpha (ERα), progesterone receptor (PR), and human epidermal growth factor receptor 2 (HER2) expression [2]. While early-stage breast cancer is curable for most patients, metastatic disease remains incurable, with resistance to currently available therapies quickly emerging, limiting survival [3].

Breast cancer therapies currently rely on traditional methods such as surgery, chemotherapies, and radiotherapy, alongside more targeted approaches, including endocrine therapies, HER2 inhibitors, cyclin-dependent kinase 4/6 (CDK4/6) inhibitors, poly (ADP-ribose) polymerase (PARP) inhibitors, and immunotherapies [4]. Oestrogen receptor-positive (ER +) is the most common subtype of breast cancer, and it can be targeted using endocrine therapies that suppress oestrogen-driven breast cancer growth. In advanced ER + breast cancer, the use of chemotherapy and CDK4/6 inhibitors is also beneficial; however, resistance to these therapies quickly renders them ineffective in many cases. HER2 + breast cancer accounts for 15% of cases and can be treated with monoclonal antibodies and tyrosine kinase inhibitors targeting HER2 [5]. Triple-negative breast cancer (TNBC) lacks ER and HER2 expression, and treatment primarily relies on chemotherapy; however, PARP inhibition can be used to exploit synthetic lethality in tumours with breast cancer susceptibility gene 1/2 (*BRCA1/2*) mutations [6]. Recently, immunotherapy has emerged as a useful approach in breast cancer; however, research is needed to identify sensitisers to improve its efficacy. Overall, resistance to therapies remains a major challenge in breast cancer treatment, highlighting the need for novel therapies and combination therapies to overcome this obstacle.

Drug discovery has, for a long time, relied on high-throughput screening approaches. Both pharmacological and genetic screens allow large numbers of targets to be tested, leading to the successful identification of new drugs and drug targets across many diseases. Clustered regularly interspaced short palindromic repeats (CRISPR)-Cas9 technology has revolutionised this process, enabling unbiased genetic screening in a pooled setting, surpassing the limitations of arrayed approaches. Furthermore, the versatility of different Cas variants enables different kinds of studies into gene function through activation or depletion and the study of non-coding RNAs (ncRNAs) [7]. The use of CRISPR technology in a variety of different *in vitro* and *in vivo* screens has uncovered a wealth of knowledge into drivers of breast cancer, potential therapies, and combination treatments. As the technology continues to develop, it will undoubtedly reveal deeper insights into the biology of breast cancer, leading to the discovery of new and more effective treatment strategies.

A vast number of studies have been conducted using CRISPR screening to expand our knowledge about how breast cancer develops, grows, metastasises, and becomes resistant to therapies. In addition, several review articles have been published with practical guides for CRISPR screening, overviews of discoveries from CRISPR-based screening approaches in cancer, and how CRISPR technology has been applied and may be used in breast cancer treatment and drug discovery [8–13]. In this review, we aim to build on this foundation by incorporating recent innovations in screening strategies, including approaches utilising organoids, *in vivo* models, and strategies targeting non-coding DNA regions, enhancer elements, and novel single-cell readout strategies. Furthermore, we provide an updated description of how CRISPR-based functional genomics continues to reshape our understanding of breast cancer. This review provides a structured overview of CRISPR screening studies grouped by key discoveries and a comprehensive table outlining technical parameters and key findings of the studies, aiming to offer a resource to guide interpretation of past findings and design of future studies.

## CRISPR-Cas9 technology

CRISPR-Cas9 technology is derived from a bacterial defence mechanism that provides adaptive immunity against viral infections. During viral infection, CRISPR incorporates short viral DNA fragments (20–50 bp) into the genome at the CRISPR locus, which is interspersed between repeating sequences of DNA. During transcription, these loci produce CRISPR RNAs (crRNAs) that, along with trans-activating crRNA (tracrRNA), guide the Cas9 endonuclease to complementary viral DNA, inducing double-strand breaks (DSBs), thereby preventing viral replication [14, 15].

The repurposing of this technology in mammalian cells offers a powerful tool to explore and interrogate gene function in different settings through targeted DNA modification. Experimentally, the guide RNA (gRNA) is an RNA composed of a crRNA, complementary to the target DNA sequence, and a tracrRNA that binds to Cas9. Often, these elements are combined, forming one single guide RNA (sgRNA). Following Cas9-induced DSBs, cells repair the damage by low-fidelity non-homologous end joining (NHEJ), resulting in the introduction of insertions and deletions, disrupting gene function through frameshift mutations [16]. DSBs can also be repaired through homology-directed repair (HDR), where template DNA can be used to introduce specific changes to DNA sequences, resulting in targeted genomic mutations [17].

The most common use of CRISPR systems is the disruption of gene expression and function by generating knockouts with gene-specific user-designed sgRNAs that induce DSBs in the DNA and cause mutations through NHEJ. Cas12 is another type of editing enzyme of the CRISPR family that uses a single crRNA as a guide to target DNA sequences, generating staggered cuts, which enhances the HDR mechanism and can be used for precise gene editing [18].

**Fig. 1:**
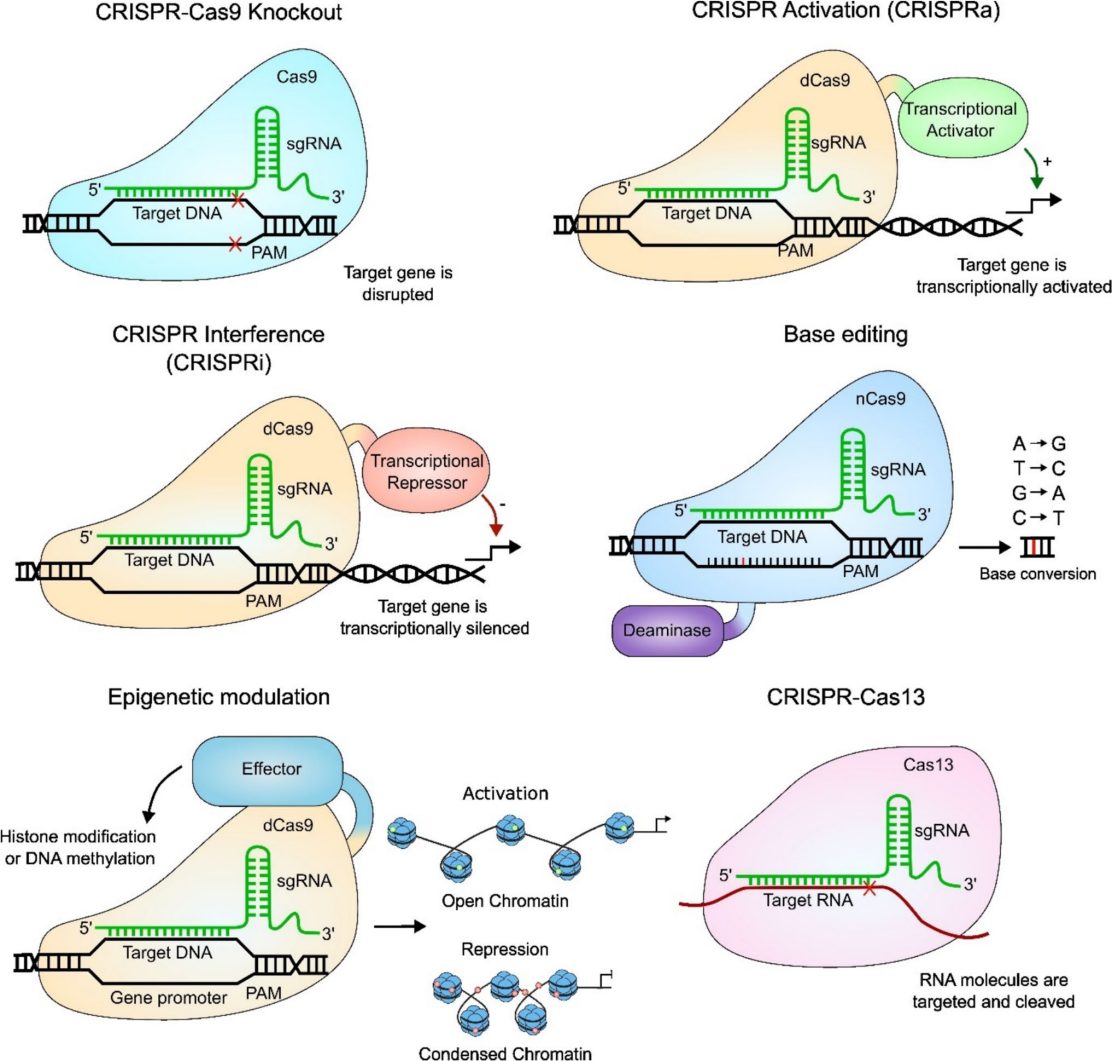
Overview of CRISPR-Cas systems and mechanisms of action. Diagram showing the different CRISPR-based systems used in functional genomics screens. The CRISPR-Cas9 system uses sgRNAs to direct the Cas9 endonuclease to target genes where it induces DSBs in the DNA, resulting in gene knockout through error-prone DNA repair mechanisms. CRISPRa uses a catalytically inactive Cas9 (dCas9) fused to a transcriptional activator. sgRNAs are designed to target upstream of the promoter region or translation start site of the target gene, inducing upregulation of the target. CRISPRi works similarly; however, it utilises a transcriptional repressor to silence target genes. Base editing through Cas9-conjugated deaminase can convert bases, inducing substitution mutations in DNA or RNA. Epigenetic modulation is also possible, where dCas9 is conjugated to an epigenetic effector that induces histone modifications or DNA methylation at the promoter of target genes, resulting in chromatin changes, altering gene transcription. Cas13 is an RNA-targeting enzyme that cleaves RNA molecules such as mRNA and lncRNA to silence gene expression without binding to DNA or inducing DNA damage

A modified Cas9 enzyme that lacks endonuclease activity, dead Cas9 (dCas9), can be used for CRISPR activation (CRISPRa) screening. Here, dCas9 is fused to a transcriptional activator, increasing transcription of genes, enabling studies to find drivers of specific phenotypes and processes. CRISPR interference (CRISPRi) works in a similar manner to CRISPRa; however, dCas9 is instead fused to a transcriptional repressor [19, 20]. Another form of transcriptional regulation of target genes via CRISPR arises through the fusion of dCas9 to epigenetic modifiers, such as DNA methyltransferase 3 A (DNMT3A), which adds methyl groups in close proximity to the target DNA, negatively regulating expression (Fig. 1) [21].

CRISPR-guided mutagenesis is another type of CRISPR-based genetic engineering in which a dead Cas nickase (dCas) is fused to a deaminase enzyme, allowing precise targeted mutagenesis without the creation of DNA breaks or the requirement for donor sequences. The deaminases are categorised into two groups, adenine base editors (ABEs) and cytosine base editors (CBEs). ABEs catalyse the A → G and T → C base conversions, while CBEs catalyse the C → T and G → A conversions [22].

On the transcriptome level, endonucleases belonging to the Cas13 family can be used to modulate gene expression. Cas13 is an RNA-guided, RNA-targeting CRISPR-Cas effector that alters gene expression through the cleavage and subsequent degradation of target mRNAs, without editing DNA [23]. In addition to transcript degradation, Cas13 can enable the base editing of target mRNAs. This is achieved using catalytically dead Cas13 (dCas13) fused to the ADAR2 adenosine deaminase domain, an enzyme catalysing adenosine to inosine deamination [24]. Recent developments of the Cas13-ADAR2 technology also allow use of the light-inducible RNA editing system *in vitro* and *in vivo* to specifically induce or inhibit the expression of targeted transcripts, or to modify post-translational modifications by specific transcript base editing [25].

Another emerging technology is prime editing, which enables precise small insertions, deletions, and substitutions in DNA without inducing double-strand breaks [26]. The technology relies on the use of a Cas9 nickase fused with a reverse transcriptase and guided by a prime editing guide RNA that directs the complex to specific DNA sites and also contains the sequence encoding the edit [26]. The Cas9 generates a single-strand break (nick) in the non-target DNA strand. The 3’ hydroxyl group then hybridises with the primer binding site, and the reverse transcriptase uses this to begin DNA synthesis on the target strand. The DNA is then repaired so that the edited DNA is incorporated into the genome [27]. Although this technology holds great promise for therapeutic applications, it can also be used in biotechnology applications, including genetic screens for the high-throughput identification of genetic variants with phenotypic effects in cells [28].

Through these different tools, many different studies have been conducted, and this review will focus on how these enzymes have been applied to screening experiments in breast cancer.

## Key considerations and design of CRISPR screens in breast cancer research

Successful implementation of CRISPR screening in breast cancer research requires the careful optimisation of several experimental parameters. These include sgRNA library design, model selection, phenotypic assay development and validation, appropriate readout strategies, and robust quality control.

The first key component of a successful CRISPR screen is the construction or selection of a sgRNA library with high on-target activity and minimal off-target effects. Several such libraries are available, including the genome-wide CRISPR knockout libraries, GeCKO and Brunello [29, 30]. These libraries include carefully selected sgRNAs based on optimised sgRNA design rules to improve the specificity of sgRNAs [31]. In addition to genome-wide CRISPR libraries, sublibraries are also available that include a specific type of target genes, such as druggable targets, proteins involved in the cell cycle, and ribosomal proteins and kinases [32, 33].

For the generation of fully customised libraries, sgRNA design tools such as CHOPCHOP, CRISPick, or E-CRISP provide user-friendly platforms to design sgRNAs with low off-target effects and high specificity to target sites [31, 34, 35]. In CRISPR screens, it is recommended to include at least four sgRNAs for each target gene, as well as positive and negative controls. Negative control sgRNAs should target loci with no effect on the phenotypic readout of the screen, whereas positive control sgRNAs need to target genes known to alter the phenotype being assessed in the screen. For example, in a CRISPR knockout screen with a readout of cell viability, sgRNAs targeting essential genes are an appropriate control, and they should be strongly depleted in the endpoint sample of the screen. The inclusion of these controls enables proper quality control to be conducted after the screening experiment is complete [11].

The widespread use of genome-wide CRISPR screens has led to the generation of extensive publicly available datasets, including data on gene essentiality in a tissue-specific context, gene interactions, drug sensitivity associations, and sgRNA efficiency across libraries. This has allowed researchers to identify the most effective sgRNAs across different experimental settings. As a result, optimised genome-wide libraries have been developed by selecting sgRNAs with superior efficacy and specificity, allowing the use of only two sgRNAs per gene, compared to traditional libraries of four or more sgRNAs per gene [36]. Smaller CRISPR libraries will have a significant advantage in resource-constrained systems where scalability is a concern, such as patient-derived organoids or *in vivo* models. These condensed libraries enable broader gene coverage in more complex systems. Additionally, when genome-wide screening is not required, sublibraries containing sgRNAs targeting a particular set of genes, such as kinases or druggable targets, may be preferable and have more translational potential.

CRISPR screens can generally be conducted in pooled or arrayed settings. In both cases, the first step involves the generation of a stable cell line expressing Cas9 or the chosen Cas enzyme. In some cases, the Cas enzyme gene is included in the library plasmid as a single vector system, but this lowers the efficiency of viral titration and transduction, due to the large sequence size of Cas genes. In arrayed screens, multi-well plates are used to transduce cells with different sgRNAs independently. Readouts will often use cell viability or apoptosis, changes in cell morphology, or expression of a fluorescent reporter [37, 38]. In a pooled CRISPR screen, sgRNAs targeting different genes are included in a plasmid library as a pool, usually carried in lentiviral vectors. The CRISPR library typically contains three to ten sgRNAs per gene for higher target efficiency, with most studies using four to six sgRNAs. HEK293T cells are commonly used to generate the lentiviral particles, with each viral particle containing a single sgRNA.

Transduction of target cells is performed with a low multiplicity of infection (MOI), typically around 0.3, to reduce the probability of multiple sgRNAs entering one cell. This generates a pool where each cell on average harbours only one sgRNA after antibiotic selection. The cells are then subjected to a selection pressure in order to enrich or deplete different sgRNAs in the final population. The sgRNAs from the initial and final population are amplified via PCR and subjected to next-generation sequencing (NGS). The sgRNA counts of each sample are then compared to identify genes involved in different processes [39]. Multiple bioinformatics tools have been developed for the data and statistical analysis, quality control, and interpretation of the CRISPR results [11].

Although viruses are the most widely used sgRNA delivery method in pooled CRISPR screens due to the high transduction efficiency, stable integration of constructs, and compatibility with many different cell types, non-viral delivery methods have also been used, particularly in arrayed screens. In these cases, electroporation or lipid-based transfection can be used for specific experimental conditions. For instance, in cells which resist transduction through lentiviral or adeno-associated viral particles, such as induced pluripotent stem cells (iPSC)-derived microglia, nucleofection is a valid alternative that has been used successfully in arrayed CRISPR screens [40]. Additionally, the immunogenicity and small insertion size of viral vectors present challenges, particularly in cell types sensitive to foreign materials, such as myeloid cells [41, 42]. Haematopoietic stem cells and some other primary cell types are resistant to viral transduction due to a variety of factors, including quiescence, low viral receptor expression, and antiviral defences [43]. Non-viral delivery of sgRNAs therefore represents an alternative strategy for CRISPR screens in cells where immunogenicity or low transduction efficiency is a concern.

Selecting a biologically relevant and technically feasible model is crucial for any CRISPR screen and requires careful consideration of all available options. Immortalised cell lines offer the highest level of scalability, ease of genetic manipulation, genetic and phenotypic homogeneity, and low cost; however, they are perhaps the least clinically relevant models. Studies often use more than one cancer cell line for the initial screen, and the top gene hits of multiple cell lines are chosen for further evaluation in more complex and clinically relevant models [44–47]. In addition, the direct comparison of more than one cell line carrying a different genetic background can provide context-dependent information. For instance, in breast cancer, some studies have conducted screens in both ER + and ER- cell lines to identify ER-dependent effects [48]. For increased relevance, patient-derived organoids/xenografts, *in vivo* models, and 3D culture models may be used, though at the cost of scalability and homogeneity [49–52]. Mouse models are increasingly used for the study of processes such as metastasis, where *in vitro* systems fail to capture the true complexity of the system [53, 54]. Moreover, *in vivo* models offer a significant advantage over *in vitro* models in studying the tumour microenvironment, as they more accurately reflect the TME observed in clinical settings [39, 50, 55, 56].

**Fig. 2:**
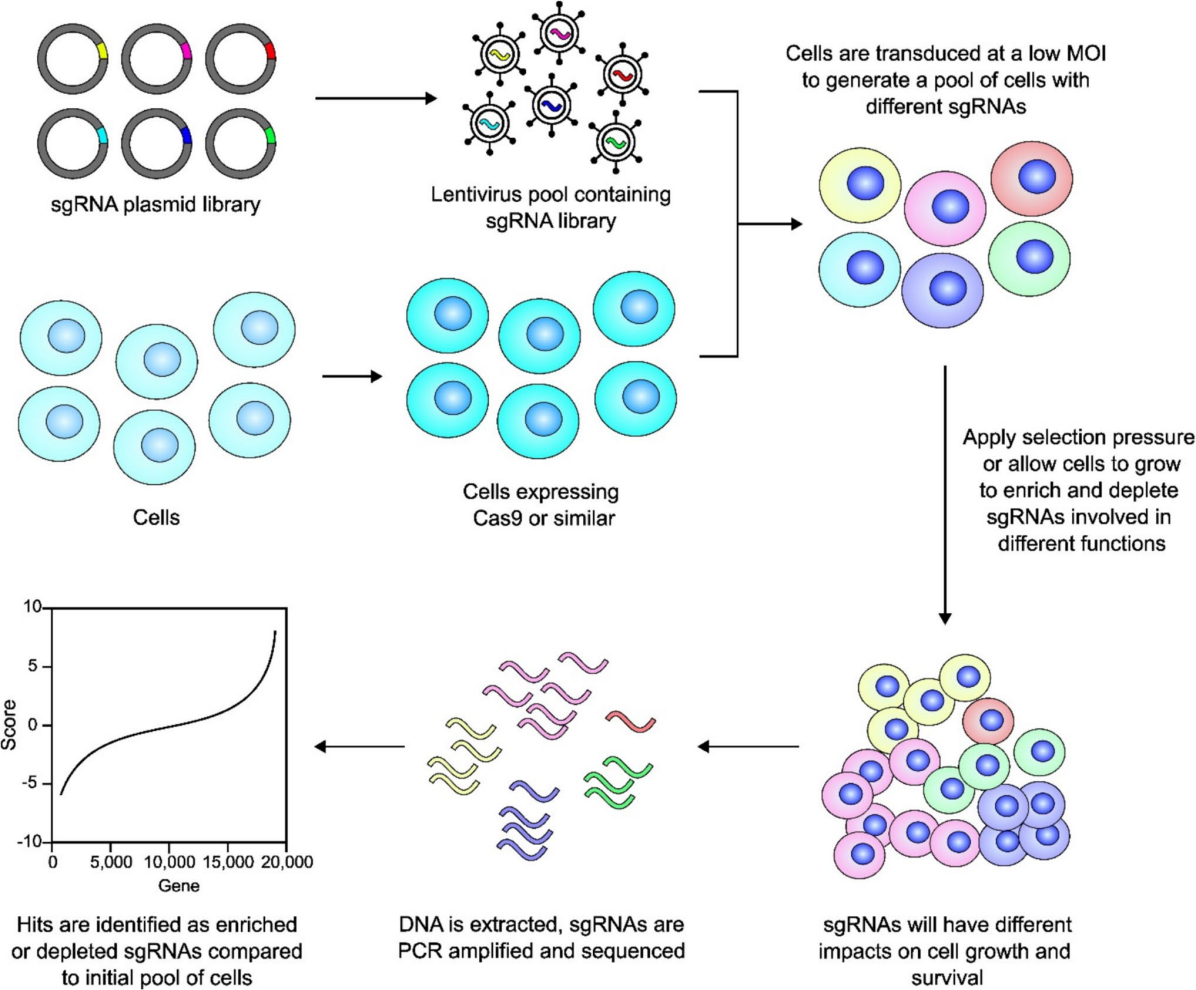
Workflow of a pooled CRISPR-Cas9 screening experiment. Schematic representation of the experimental workflow of a pooled CRISPR-Cas9 screen. Initially, a genome-wide or targeted sgRNA library is amplified and packaged into lentiviral particles through transfection of HEK293T cells with the library and packaging plasmids. The lentivirus is harvested, and the viral titre is determined. Cas-expressing cells are then infected with the lentiviral library at a low multiplicity of infection (MOI), increasing the probability that each cell is transduced with a single sgRNA. The resulting heterogeneous pool of cells is then exposed to a selection pressure, either *in vivo* or *in vitro*, to enrich or deplete specific sgRNAs based on their impact on cell phenotype. Genomic DNA is then extracted from the final and initial cell populations, and sgRNAs are amplified by PCR then subjected to next-generation sequencing. This quantifies the abundance of each sgRNA, enabling the identification of genes associated with resistance to drugs, growth, metastasis, or other phenotypic responses

The choice of phenotypic assays and screen readouts are both key considerations when designing a CRISPR screen, as these determine the type of biological insight gained from the experiment. Pooled CRISPR screens allow for a range of different selection pressures to be applied, and these can be conducted either *in vitro* or *in vivo* (Fig. 2). A simple screen format involves transducing cells and allowing them to proliferate under normal conditions to identify genes whose disruption induces cell death or confers a growth advantage. These screens can also be conducted in the presence of a selective agent, such as a chemotherapy drug, endocrine therapy, or targeted therapy, to find synthetic lethal genes or promoters of resistance to certain treatments. CRISPR screens can also broadly be categorised into positive selection or negative selection approaches. Positive selection screens aim to identify genes that, when disrupted, provide a beneficial phenotype or increased survival under the selective pressure applied. For example, following transduction of the pooled sgRNAs, cells are treated with a chemotherapeutic drug, and cells that survive the selection period will contain sgRNAs that confer resistance to the treatment. In a negative selection, genes that are essential for cell survival get depleted, and their sgRNAs become depleted in the samples following selection [11, 38].

The most common readout of cell viability or drug resistance screens is the sgRNA enrichment or depletion in the endpoint samples, which is identified by bulk NGS of the initial and endpoint samples. Well-established pipelines using tools such as MAGeCK are used to analyse the NGS results and allow the identification of depleted or enriched sgRNAs in the samples [57]. Proper quality control is essential before drawing conclusions from CRISPR screening experiments. Sufficient reads per gRNA are needed to give sufficient representation. Depletion of essential genes and selection of positive control genes based on their expected effect on the phenotype are also key indicators of the success of a screen. Negative control genes should be unaffected by the experiment. Furthermore, biological replicates would be expected to show similar changes in sgRNA representation [11].

More complex models also exist where regulators of genes or signalling pathways can be found by performing a pooled CRISPR screen in reporter cell lines, which are then subjected to fluorescence-activated cell sorting (FACS) for high or low presence of key proteins or activity of pathways. The sorted populations are then analysed by NGS to identify enriched or depleted sgRNAs, which ultimately uncovers new regulators of the studied signalling pathway [58–60].

More complex readouts, including single-cell RNA sequencing or spatial imaging, provide richer and multidimensional data, but demand a higher budget and expertise. Advancements in both single-cell RNA sequencing and CRISPR technology enable the target-specific analysis of transcriptome profiling on a cellular level [57]. Perturb-seq is an innovative approach where single-cell RNA sequencing is combined with pooled CRISPR screening to achieve high-content measurement of the effects of sgRNAs on cells [61]. This technology has already shown promise in immune-based screens, where complex changes in gene expression are far more important readouts than pooled phenotypic effects. Another similar approach is called CROP-seq, which builds on Perturb-seq by enabling direct sequencing of the sgRNA [57].

The development of multiplexed phenotypic readouts for CRISPR screening allows the simultaneous study of protein or RNA expression and localisation within the cells, cellular morphology, and cell–cell interactions, coupled with specific gene perturbations. CRISPRmap consists of a barcoded sgRNA library that can be applied to *in vitro* cell culture and tissue samples obtained from *in vivo* models. This technology allows the detection of cells that have received different sgRNAs, while simultaneously proteins or RNA transcripts are detected *in situ*, giving valuable information about protein abundance or cellular localisation [62].

Another innovative advancement in CRISPR screening technology is the use of Pro-Code protein barcodes [63]. In conventional pooled CRISPR screens, the identity of the sgRNA in each cell is determined by DNA sequencing. Although powerful, this technique relies on alterations across the population of cells and does not provide single-cell resolution. Additionally, using protein expression as a readout can be challenging. In contrast, the Pro-Code system uses combinatorial protein epitopes to serve as cell surface barcodes, which enables the sgRNA identity to be assessed at the protein level in the cells. Each sgRNA vector is engineered to encode a unique combination of epitopes (such as HA or FLAG), forming a barcode that is detectable by cell surface labelling with antibodies. To analyse the effect of the sgRNA on cells, the cells are then stained with metal-conjugated antibodies and analysed by mass cytometry (CyTOF) allowing simultaneous detection of up to 45 Pro-Code barcodes and phenotypic marker proteins [64]. This single-cell resolution assay can allow protein level readouts, and the authors showed it could be used to identify genes important for antigen-dependent immune editing of breast cancer cells [63]. More specifically, they used Pro-Code CRISPR vectors to screen for genes involved in breast cancer resistance to T cell killing, revealing Psmb8 and Rtp4 as key proteins in antigen-dependent immune editing. The technology will have powerful implications for CRISPR screens in cancer, particularly where the expression of proteins is the optimal readout, such as in immunology. However, when the expression of only a small number of proteins is used as a readout, reporter-based CRISPR screens using fluorescence sorting as a readout are sufficient and more cost-effective, while enabling the identification of regulators of genes [11].

## Applications of CRISPR screening in breast cancer

CRISPR technology has emerged as a powerful tool, supporting breast cancer research by enabling high-throughput screening to uncover genes involved in diverse processes of tumourigenesis and response to therapies. A summary of the CRISPR screening experiments conducted in breast cancer research and their main findings is presented in Table 1.

**Table 1:** Details of studies into breast cancer using CRISPR-based screening approaches

Study objective	Library	Screen details (cell line, *in vitro*/*in vivo*, MOI)	Selection/model	Key findings	Reference
Identifying drivers of breast cancer					
Exploring tumour suppressors and oncogenes in TNBC	GeCKOv2 (19,050 genes, 1000 control sgRNAs)	SUM159PT cells *in vivo*, MOI = 0.3	Subcutaneous injection in NSG mice, proliferation based	Inhibition of YAP and mTORC1/2 reduce tumourigenesis in TNBC	[50]
LncRNA activators of AKT in breast cancer	LncRNAs with 5 sgRNAs per lncRNA and 10 negative controls	MCF7 cells *in vitro*, MOI < 1	AKT reporter was in the cells conferring resistance to puromycin	AK023948 interacts with DHX9 and p85, increasing AKT activity	[65]
Screen for lncRNAs in human cancer	3913 sgRNAs targeting the cryptic ORFs identified through ribo-seq	MCF7 cells *in vitro*	Cell growth *in vitro* for 21 days	GATA3-interacting cryptic protein (GT3-INCP) drives breast cancer growth	[66]
Identification of gene targets at breast cancer risk loci	184 level 1, 678 level 2, 371 TWAS identified genes, 605 background genes with 5 sgRNAs per gene, 1000 negative and 960 positive control sgRNAs	Six immortalised mammary epithelial cell lines *in vitro* then secondary screen *in vivo*	Cell growth *in vitro* and *in vivo*	Identification of 20 genes that promote breast cancer	[71]
Regulators of alternative splicing in breast cancer	954 sgRNAs targeting 159 RNA-binding proteins, 12 control sgRNAs and 42 positive control sgRNAs	MCF10 cells *in vivo*, MOI = 0.3	Subcutaneous injection in NOD/SCID mice, proliferation based	PHF5A promotes migration, survival and metastasis	[51]
Exploring Hippo signalling in breast cancer	GeCKOv2 library	MCF10a cells with dox-inducible Strep-tagged YAP 5SA allele and a turboRFP reporter. Expression is driven by a small promoter fragment containing TEAD-binding sites of the YAP target gene, CTGF	Doxycycline followed by sorting for RFP	TRPS1 globally regulates YAP-dependent transcription, repressing YAP functions	[69]
Exploring CTCF anchor sites in ER-driven BC proliferation	Custom CTCF-binding element (CBE) library targeting sites close to ERα-bound enhancers and DNA loop formation sites	MCF7 and MDA-MB-231 *in vitro*	Cell proliferation (20 days)	CTCF-mediated chromatin structures are necessary for ERα to promote breast cancer proliferation	[48]
Identifying drivers of metastasis in CTCs *in vivo* in ER + BC	70,290 sgRNAs, covering all 23,430 human coding isoforms (Genome-wide)	Patient-derived CTC cells (Brx-82 and Brx-142) *in vivo*	Injection into mouse tail vein, formation of metastases	Overexpression of RPL15, a large ribosomal subunit component, strongly increases formation of distal metastases	[52]
*In vivo* screen for metastatic progression	Human GeCKO library	CTC-derived BR16 cells *in vivo*, MOI = 0.3	Injection in mammary fat pad of NSG mice, allowed to grow for 22–28 weeks	CTC intravasation is sensitive to PLK1 inhibition	[72]
CRISPR screen for regulators of EMT	Genome-wide sgRNA library then EPIKOL library	HMLER cells *in vitro*, MOI < 1	Cells grown *in vitro* then sorted by MACS and FACS for mesenchymal populations	PRC2 and KMT2D-COMPASS regulate distinct EMT trajectories	[73]
Druggable genome in TNBC	LentiCRISPR V2 pooled library of 2240 genes targeted by 5 sgRNAs each	D492 and D492M cells *in vitro* treatment, MOI = 0.2	1 µm 5‐FU or 5 nm everolimus drug for 2–4 weeks	Loss of EGFR and fatty acid synthase (FASN) sensitised cells to 5-FU and everolimus	[87]
Exploring lipid metabolic gene drivers of metastasis in TNBC	Lipid metabolic genes library targeting 678 metabolic genes with 4499 sgRNAs and 431 negative controls	MDA-MB-231LM2/Cas9/luciferase-GFP cells *in vivo*	Implantation in nude mice, formation of metastases	NSDHL drives metastasis in TNBC through TGFβ signalling	[53]
Dissecting transcriptional regulatory networks with genome-scale and single-cell CRISPR screens	Multiple—TRE-specific library with 7992 sgRNAs for ESR1 and 26,263 sgRNAs for CCND1. GERCAR library (80,254 sgRNAs)	MCF7 cells *in vitro*, MOI = 0.3	Proliferation or FACS	Discovery of TREs involved in ER oncogenic activity and mapping of regulatory networks	[74]
RNA-binding proteins in breast cancer	10 sgRNAs for each of 1078 RBPs, 628 sgRNAs positive controls and 1058 non-targeting sgRNAs as negative controls	HMEC cells expressing MYC-ER *in vitro*, MOI = 0.3	Tamoxifen-induced MYC activity	Loss of YTHDF2-dependent mRNA degradation induces apoptosis in TNBC	[70]
Exploring pseudogenes in luminal A breast cancer with CRISPRi	Custom—11,915 sgRNAs, including 568 sgRNAs targeting 71 core essential genes, 267 sgRNAs targeting AAVS1 and 83 non-targeting sgRNAs	MCF7 stably expressing dCas9-KRAB *in vitro*	Cell proliferation (21 days)	MGAT4EP interacts with FOXA1, enhancing promoter binding of FOXA1, increasing expression of FOXM1	[75]
Combinatorial screening with scRNA sequencing for tumour suppressor networks in BC	CROP-seq library containing 72 pairwise sgRNA combinations	MCF10A cells *in vitro* and *in vivo*	*In vitro* and *in vivo* proliferation. Orthotopic inoculation of nude mice	Cooperating tumour suppressor genes that synergise in promoting tumourigenesis show transcriptional epistasis, but noncooperating TSGs do not show epistasis	[76]
LncRNA drivers of breast cancer	Custom CRISPR-Cas13 guide RNA library (targeting 1864 lncRNAs)	MCF7 and MDA-MB-231 cells *in vitro*, MOI = 0.3	Cell proliferation	Loss of KILR promotes growth through increased RPA1	[67]
miRNAs involved in breast cancer tumour-initiating cell growth	GeCKOv2 library	MCF7 and HCC1395 cells *in vitro*, MOI = 0.3	Cell proliferation and tumour sphere formation	miR-4787-3p inhibition reduces tumour sphere formation	[77]
Exploring factors governing CTC survival	human CRISPRa pooled library Set A	Patient-derived CTC organoids *in vitro*	Withdrawal of NRG-1 from NRG-dependent CTC-derived organoids	NRG1 and FGFR1 signalling work to sustain CTC growth and survival. Combination treatment could be an effective therapy	[49]
Screening for epigenetic vulnerabilities	EPIKOL (Epigenetic knockout library) 719 chromatin-related genes, 25 genes from other families, 35 essential genes	MDA-MB-231, SUM149PT and SUM159PT and HMLE cells *in vitro*, low MOI	Cell proliferation	Library validation, growth regulation by SS18L2 and members of the NSL complex (KANSL2, KANSL3, KAT8) in TNBC	[144]
Screening for drugs against TNBC	Genome-wide CRISPR-guide RNA library of unique sgRNAs targeting 20,000 genes	BT-549 and MDA-MB-468 cells *in vitro*	Cell proliferation (21 days)	UBE1 is a druggable target in c-MYC high TNBC	[44]
Identifying drivers of metastasis	Small custom library of 440 genes related to metastasis	MDA‐MB‐231 and LM2‐4175 cells *in vivo*	Injection of cells in mouse tail vein for metastasis. NGS of lung and liver metastases	RhoV promotes metastasis in TNBC	[145]
Screening for RNA-binding proteins involved in TNBC	8667 sgRNA sequences (7667 sgRNAs targeting 1114 genes + 1000 control sgRNAs) RBP library	DCIS and CA1a cells *in vivo*, MOI = 0.3	Subcutaneous growth in mice	SNRPC promotes TNBC progression	[146]
Screening for regulators of dormancy and recurrence	Custom sgRNA library targeting 95 candidate genes with 4–5 sgRNAs focused on ECM-related genes	Her2-dependent Cas9 cells *in vivo*, MOI = 0.3	Subcutaneous growth in mice, selection of dormancy-associated genes	B3GALT6 regulates dormant, therapy-refractory residual tumour cells fitness	[147]
Screening for regulators of anoikis resistance in TNBC	GeCKOv2 CRISPR libraries	MDA-MB-231 cells *in vitro*	Cell growth using normal or ultra-low attachment conditions	PTPN14 regulates anoikis in TNBC	[148]
Screening for long tail drivers of breast cancer	sgRNA library targeting top predicted tumour suppressor genes	MCF10F cells *in vivo*, MOI = 3 to facilitate delivery of multiple sgRNAs per cell	Cells were implanted orthotopically in immunocompromised NOD-SCID mice	Far upstream binding protein 1 (FUBP1) disrupts cellular differentiation, cooperating with tumour suppressors to drive cancer growth	[78]
Screening for mediators of Beclin 1 function	Human Brunello CRISPR knockout pooled library	MCF7 *in vitro* with and without Beclin 1 expression, MOI = 0.5–0.65	Cell growth *in vitro* with and without Beclin 1 expression	Beclin 1 promotes membrane localisation of E-cadherin	[81]
*In vivo* genome-wide screening identifies collaborating tumour suppressor genes in Trp53 deficient tumours	Mouse genome-wide CRISPR knockout library	Mouse mammary cells (CD29^Hi^/CD24^+^) obtained from preneoplastic BALB/c‐*Trp53*^+/–^ mammary glands, transplanted in fat pads of BALB/c mice	Tumour growth from primary mouse epithelial cells with *Trp53* haplo-insufficiency	Loss or mutation of *Axin1* or *Prkar1a* promote tumour growth synergistically with *Trp53* haplo-insufficiency in breast cancer	[84]
Super-enhancer drivers of TNBC	3875 sgRNAs	Cell growth *in vitro* using various TNBC cell lines, MOI = 0.3	Cell growth *in vitro*	BMP and activin membrane-bound inhibitor (BAMBI) drives breast cancer growth	[79]
Screening of transcribed super-enhancers in TNBC	Arrayed CRISPRi screen targeting 30 transcribed super-enhancers	MDA-MB-436 cells expressing dCas9-KRAB	Transcriptome analysis by RNA sequencing, 3 days after sgRNA transfection	Mapping of differentially expressed genes coupled with the transcribed super-enhancers reveals the intricate relationship of eRNA and gene regulation in TNBC	[80]
Base editing screen for DNA damage response variants	DNA damage response targeting library	MCF10A-BE3, MCF7-BE3 and HAP1-BE3 *in vitro*, MOI < 0.4	Cell growth *in vitro* in the presence of cisplatin, olaparib, doxorubicin or camptothecin	Identified variants of DNA damage response genes involved in altered cellular fitness upon DNA damage	[82]
PRIME editing screen for DNA variants	Custom library of reference or alternative alleles	MCF7 *in vitro*, MOI = 0.3	Cell growth *in vitro*	Characterised effect of SNPs on cell fitness	[83]
Chemotherapy resistance					
Identifying regulators of paclitaxel resistance in TNBC	GeCKOv2 library	SUM159PT cells *in vitro* and *in vivo*, MOI = 0.3	Treatment of cells *in vitro* with paclitaxel (10 nM). Subcutaneous injection in NSG mice, treatment with paclitaxel (15 mg/kg; intraperitoneal injection) once/week for 3 weeks	FGR2 activation sensitises TNBC tumours to paclitaxel	[86]
Screening DUBs for resistance to YM155	Custom DUB arrayed screen	MCF7 cells *in vitro*	Treatment with YM155	USP32 conveys resistance to YM155	[88]
Screening for paclitaxel resistance proteins	Kinome-wide pooled library	MDA-MB-231 and MDA-MB-468 breast cancer cells *in vitro*	Treatment with paclitaxel at 20% inhibitory concentration	PLK1 silencing inhibits cancer growth and regulates paclitaxel resistance	[45]
Sensitisers to rapamycin and paclitaxel	GeCKOv2 library	MCF7 cells *in vitro*, MOI = 0.2–0.4	Transwell invasion assays to collect invasive and non-invasive cells, followed by *in vivo* validation screen	TTC17 loss drives metastasis. Combining paclitaxel with rapamycin inhibits TTC17 depleted cell growth	[149]
Radiotherapy resistance					
Sensitisers of radiotherapy in brain metastases	GeCKOv2 library	MDA-MB-231-Br-HER2 (231BR) cells *in vitro*	Exposure of cells to 10 Gy radiation followed by cell recovery. Surviving cells were collected	LRRC31 is a radiosensitiser that works through DNA damage response	[89]
Synthetic lethality with radiotherapy	Toronto KnockOut (TKOv3) sgRNA library	MCF10A cells *in vitro*	Exposure of cells to 20 Gy radiation	BCL2L1 inhibition is synthetically lethal with radiotherapy	[90]
ER + breast cancer					
Identify mechanisms of resistance to endocrine therapies in ER + breast cancer	GeCKOv2 library	MCF7 and T47D cells *in vitro*	Culture of cells in hormone depleted media and treatment with oestrogen	Oestrogen drives C-terminal SRC kinase (CSK) expression. Low CSK promotes p21-activated kinase 2 (PAK2) activation, driving growth	[46]
Epigenome CRISPR knockout screen to find fulvestrant resistance mechanisms	11 K sgRNA library targeting epigenome (914 genes)	MCF7 cells *in vitro*	Fulvestrant (100 nM) treatment for 2 weeks	Loss of ARID1A plays a key role in loss of luminal identity in ER + breast cancer cells	[95]
Oestrogen therapy sensitisers	Human Brunello CRISPR knockout pooled library	HCC-1428/LTED cells *in vitro*, MOI = 0.3	Oestrogen treatment	PARP inhibition can be used synergistically with oestrogens to suppress growth	[98]
Tamoxifen resistance in ERα breast cancer	Human Brunello CRISPR knockout pooled library	MCF7-V cells *in vitro*	Drug treatment with E2, 4-OHT, ICI, db-cAMP	PAICS inhibition sensitises cells to tamoxifen	[93]
Tamoxifen resistance in ERα breast cancer	Brunello sgRNA library in 110 LentiCRISPR v2	MCF7 cells *in vitro*, MOI = 0.2, *in vitro*	Tamoxifen treatment (12 µM) for 72 h	Tafazzin increases resistance to tamoxifen by altering phospholipid composition	[94]
Screening for genes involved in endocrine resistance	Human Brunello CRISPR knockout pooled library	T47D cells *in vitro*, MOI = 0.3	Treatment with OHT (1 µM) for 3 weeks in oestrogen rich media	SMAD4 depletion induces endocrine resistance	[150]
Genome-wide CRISPR screen for mediators of endocrine therapy resistance	Genome-wide human gRNA library	MCF7 cells *in vitro*, MOI = 0.3	Tamoxifen or fulvestrant treatment for 26 days	ARID1A and other SWI/SNF complex components are required for response to endocrine therapies	[99]
HER2 + breast cancer					
Regulators of anti-HER2 therapy resistance	GeCKOv2	SKBR3 trastuzumab-resistant cells *in vitro* and *in vivo*, MOI = 0.3	Treatment with trastuzumab (1 µM for *in vitro* screening; 20 mg/kg for *in vivo* screening)	FGFR4 upregulation promotes anti-HER2 therapy resistance in HER2 + breast cancer	[100]
Screening for autophagy regulators involved in metastasis	Custom CRISPR-Cas9 KO library containing 1026 single guide RNAs (sgRNAs) targeting 171 mouse genes involved in autophagy	N148 cells from MMTV-Neu mice, MOI < 0.3	Orthotopic transplantation in nude mice, sequencing of lung metastases	p47 suppresses HER2 + breast cancer metastasis	[54]
BRCA-mutant breast cancer					
Identifying factors involved in PARPi resistance	DNA damage response and genome-wide CRISPR libraries	KB1P-G3 cells used for DDR library, mouse embryonic stem cells and SUM149PT cells used for genome-wide library, *in vitro*	Treatment with PARPis: olaparib alone, olaparib + AZD2461, and talazoparib	CTC1-STN1-TEN1 (CST) complex component deletion induces PARPi resistance	[102]
Finding synthetic lethal genes with PARP inhibition	GeCKO library	SUM149PT cells *in vitro*	Treatment with PARP inhibitors olaparib, talazoparib (BMN673) or AZD2461	C20orf196 and FAM35A inactivation promotes PARPi resistance	[103]
Finding synthetic lethal genes with BRCA1 loss	Custom druggable library	BRCA-mutant (MDA-MB-436, SUM149PT), and BRCA WT (MDA-MB-231) *in vitro*	Cell proliferation	MEPCE loss is synthetically lethal with BRCA loss	[105]
Finding synthetic lethal genes with BRCA deficiency	Mouse GeCKOv2 sgRNA library	Brca1-deficient MEFs *in vitro*	Serial passaging	Cullin-5 loss promotes tumour growth through CREB1-CCL2 through the TME	[151]
PARP1 point mutations screen	Genome-wide mutagenesis mouse sgRNA library	Mouse ES/SUM149 cells, MOI = 0.3 *in vitro*	PARPi treatment (talazoparib) 25 nM (mouse cells) or 100 nM (SUM149 cells)	Parp1 mutations cause PARPi resistance	[104]
Identification of genes involved in PARPi hypersensitivity	Custom library targeting 197 functional domains of 179 chromatin regulators	CAPAN-1, SUM149PT and UWB1.289 *in vitro*, MOI = 0.3–0.4	Cell growth in the presence of 10 nM olaparib for 14 population doublings	ALC1/CHD1L loss restores sensitivity to PARP inhibition in BRCA-mutant breast cancer	[106]
CDK4/6 inhibitors					
CDK4/6 inhibitor sensitisation in TNBC	GeCKOv2 library	SUM159PT cells, MOI = 0.3	Subcutaneous injection of cells in NSG mice, treatment with palbociclib (30 mg/kg), 1/day 5 days/week for 23 days	TGFβ3 inhibition sensitises TNBC to palbociclib	[111]
Regulation of senescence in CDK4/6 inhibitor treated breast cancer	GeCKOv2 library	MCF7 cells *in vitro*, MOI = 0.2–0.5	Treatment with palbociclib (200 nM) for 14 days	coagulation factor IX (F9) knockdown prevents cell cycle arrest in MCF7 cells treated with Palbociclib	[107]
Synthetic lethal genes with CDK4/6 inhibitors	Transcription factors and epigenetic molecules	MCF7 cells *in vitro*, MOI = 0.3	Treatment with abemaciclib (20 nM) for 3 weeks	GATAD1 loss is synthetically lethal with CDK4/6 inhibitors	[108]
Synthetic lethal genes with RB loss	Human Brunello CRISPR knockout pooled library	T47D_WT and _RBKO cells *in vitro*	Cell growth *in vitro* comparing wildtype and RB knockout cells	PRMT5 inhibition blocks G1/S transition in RB KO cells	[110]
CDK4/6 inhibitor sensitisation	GeCKOv2 library	MCF7 cells *in vitro*	Treatment with palbociclib (2 nM) for 12 days	SEMA3F knockdown increases viability in presence of CDK4/6 inhibitors	[109]
Screening for CDK4/6 inhibitor sensitisers	Human sgRNA library containing 87,897 sgRNAs	MCF7 cells *in vitro*	Treatment with palbociclib (500 nM) for 2 weeks	GPX4 inhibition increases CDK4/6 inhibitor response	[152]
Screening for sensitisers to CDK4/6 inhibitors	Human CRISPR/Cas9 KO Pooled Library (117,587 sgRNAs, and 3842 sgRNAs targeting controls)	T47D cells, sensitive and resistant to palbociclib, MOI = 0.1	Treatment with palbociclib (100 nM or 1 µM) for 10 doublings	CDK7 inhibition combined with endocrine therapy can overcome treatment resistance	[153]
METTL14 is a key driver of CDK4/6i resistance in ER + BC	Human pooled CRISPR activation library (SAM v2)	T47D cells *in vitro*, MOI = 0.3	Treatment with abemaciclib for two weeks, NGS analysis to identify hits that mediate CDK4/6i resistance	METTL14 promotes E2F translation and cell cycle progression. Combined CDK4/6i and METTL14 inhibition results in re-sensitisation to CDK4/6i therapies *in vitro* and *in vivo*	[114]
Targeted therapies					
TTK inhibitor resistance mechanisms	Toronto Human Knockout Pooled Library [154]	MDA-MB-468, MDA-MB-436, MDA-MB-231 cells *in vitro*, low MOI	CFI-402257 treatment *in vitro* (between IC60–IC90 concentrations)	APC/C component knockout promotes resistance to CFI-402257	[116]
ADC therapy CRISPR screen	Genome-wide library	Ramos cells *in vitro*, MOI = 0.3–0.4	Treatment with 2 nM anti-CD22-Asp-PEG2-maytansine ADC for 48 h × 4 times over 3 weeks with recovery	C18ORF8/RMC1 is a novel regulator of ADC toxicity	[117]
Mediators of EGFR inhibitor sensitivity	GeCKOv2 library	BT20 cells *in vitro*, low MOI	Treatment with erlotinib (10 µM) for 3–4 population doublings	ELP depletion confers sensitivity to EGFR inhibition	[118]
Combinatorial therapies with MLN8237	Custom kinase-targeting library	MDA-MB-231 cells *in vitro*, MOI = 0.3	Treatment with MLN8237 (150 nmol/L, specifically to inhibit Aurora‐A activity)	Haspin is synthetically lethal with MLN8237	[121]
Overcoming resistance to PI3Kα inhibitors	Genome-wide (150,000 sgRNAs targeting over 19,000 human protein-coding genes), 8 sgRNAs per gene	MCF7 and T47D cells *in vitro*, MOI = 0.3	Treatment with alpelisib (1 µM) or taselisib (60 nM) to inhibit PI3Kα	mTOR inhibition can overcome resistance to PI3Kα inhibitors	[47]
Regulators of immune escape in TNBC	Disease-related immune genes (12,000 sgRNAs targeting 2796 genes, 4 sgRNAs per gene and 816 non-targeting control sgRNAs)	4T1 cells *in vivo*	Subcutaneous transplantation of cells in immunocompetent BALB/c or immunodeficient mice	Galectin-2 regulates immune escape in TNBC	[127]
Regulators of MEK inhibitor resistance	GeCKOv2 library	BT549 cells *in vitro*, MOI = 0.3	Treatment with AZD6244 (1 µM) for 7 days	PSMG2 inhibition increases the efficacy of the MEK inhibitor AZD6244	[122]
Exploring sensitisers to SRC therapies	Pooled lentiviral genome-wide CRISPR-SpCas9 TKOv3 library targeting 18,053 protein-coding genes (4 sgRNAs/gene)	MDA-MB-231 cells *in vitro*, MOI = 0.36	Treatment with EC20 concentration of bosutinib (0.9 µmol/L) for 3 days for five rounds over 20 days	Integrin-Linked Kinase loss sensitises cells to SRC inhibitors	[123]
Synthetic lethal interactions for PARP10	Brunello Human CRISPR knockout pooled lentiviral library	MCF10A wildtype, MCF10A-TREPARP10, HeLa wildtype and HeLa-PARP10KO cells *in vitro*, MOI = 0.4	Cell proliferation	PARP10 impacts recruitment of ATM to DNA. CDK2-Cyclin E1 is essential in PARP10 knockout cells	[155]
Overcoming PI3K-AKT inhibitor resistance	Yusa Human CRISPR library V1, 18,009 genes with 90,709 sgRNAs	EVSA-T, HCC70 and ZR-75–1 cells in vitro, MOI = 0.3	Treatment with AZD8186 (250 nM) and capivasertib (1 µM) for EVSA-T; AZD8186 (100 nM) and capivasertib (500 nM) for HCC70; AZD8186 (50 nM) and capivasertib (500 nM) for ZR-75–1 for 12–21 days	Mcl-1 loss enhances apoptosis with AKTi and PI3Kβi	[156]
Screening for CTR1 upstream kinases	Kinome-wide CRISPR library	GFP-fusion CTR1 and separated expression of RFP HEK293T cells *in vitro*	FACS	AMPK phosphorylates CTR1, resulting in protein stabilisation	[157]
Screening for synergistic kinase inhibitors to combine with transcriptional CDK inhibitors	Brunello pooled human CRISPR knockout library	Hs578T cells *in vitro*, MOI = 0.1	Treatment with THZ531 (0.1 µM)	THZ531 (CDK12/13 inhibitor) synergises with ABCG2 kinase inhibition	[158]
Screening for mechanisms of necrosis with BHPI	TKOv3 library (genome-wide)	T47D cells *in vitro*, MOI = 0.3	Cells were grown in 100 nM BHPI necrosis-inducing preclinical agents	TRPM4 is downregulated in cells resistant to BHPI and ErSO	[159]
Screening for sensitisers to EGFR inhibitors	TKOv3 genome-wide library	CAL51 cells *in vitro*, MOI < 0.2	Treatment with gefitinib (750 nM) and THZ531 (100 nM) for 3 weeks	CDK12/13 targeting synergises with EGFR inhibition	[119]
Screening for synthetically lethal genes with MYC-driven BC	TKOv3 genome-wide library	10A.PE isogenic control and and MYC-driven 10A.PM human breast cancer cells *in vitro*, MOI = 0.3	Cell growth for 22 days	Topoisomerase 1 inhibition induces synthetic lethality with MYC-driven cancer	[160]
Synthetic lethality with AKT/PI3K inhibitors	Genome-wide library	SUM159 and H1047L cells *in vitro*	Treatment with PI3Kα inhibitor BYL719 (0.4 µmol/L) or AKT inhibitor GDC-0068 (3 µmol/L)	TNBC cells are vulnerable to treatment with AKT inhibitors combined with cholesterol biosynthesis targeting pitavastatin	[161]
Resistance to NOTCH inhibitors	GeCKOv2 library	MB157R cells *in vitro*, MOI = 0.3–0.4	Treatment with γ-secretase inhibitor GSI (10 µM) for 14 days	GSI-PTX and DTB-PTX combination therapy reduces tumour growth and metastasis	[162]
Screening for sensitisers to KDM5B inhibitors	Human CRISPR knockout library (H3)	SUM149 or SUM149CR cells *in vitro*, MOI = 0.3	Treatment with C70 (10 µM) for 10 cell doublings	ZBTB7A depletion sensitises cells to KDM5 inhibition	[163]
Screening for synthetic lethal interactions with BET bromodomain inhibitors	Human pooled lentiviral genome-wide library	SUM149 and SUM159 cells *in vitro*, MOI = 0.3	DMSO or JQ1 (500 nM and 1 µM for SUM159; 100 nM, 200 nM, 400 nM, 800 nM for SUM149; 20 µM for SUM159R; 10 µM for SUM149R cells)	CDK4 and BRD2 are synthetically lethal with BET bromodomain inhibitors	[124]
Combinatorial CRISPR screen identifies FYN and KDM4 as synergistic drug targets in TNBC	Combinatorial CRISPR library containing sgRNAs for 76 tyrosine kinases	MDA-MB-231 cells *in vitro*, MOI = 0.3	Cell proliferation *in vitro*	Combinational inhibition of KDM4 and FYN under TKI treatment results in higher therapeutic effects *in vitro* and *in vivo* in TNBC	[125]
Immunotherapies					
CD8 + T Cell genome-wide screen in TNBC	Custom mouse genome-wide library (128,209 gene-targeting sgRNAs and 1000 non-targeting controls	Mouse-derived T cells used *in vivo*	Mouse-derived T cells were extracted, infected then re-injected into mice. Tumour infiltration was assessed	DHX37 knockout in CD8 + T cells enhances anti-tumour response	[55]
Screening for kinases to enhance anti-cancer T cell activity	Arrayed screen of 25 T cell receptor-driven kinases	Mouse primary T cells *in vitro*	Arrayed screen without selection, restimulation and expansion of T cells followed by flow cytometry	p38 regulates T cell expansion, stemness and metabolic fitness	[126]
Exploring macrophage infiltration genes and cancer immunotherapy targets	Murine lentiviral CRISPR-Cas9 knockout (MusCK) library consisting of 5 sgRNAs for each of 4500 genes involved in tumourigenesis and immune modulation, with secondary screen of 79 hits from primary screen (8 sgRNAs per gene)	4T1 TNBC mouse cells expressing membrane-bound ovalbumin to enhance immune response, used *in vivo*	Syngeneic BALB/c mice *in vivo* model, compared immunocompetent with nude mice	Cop1 deletion in BC reduces macrophage recruitment to tumours, improving anti-tumour immunity and improving ICB response	[56]
Anti-PD-1 therapy sensitisation	The genome-wide Brunello sgRNA library	MDA-MB-231 cells *in vitro*, MOI = 0.2	Treatment with nivolumab (10 µg/ml) and co-culture with primary human lymphocytes for 6 days	NEDD8 loss increases sensitivity to Nivolumab	[129]
Macrophage phagocytosis enhancers	LentiCRISPRv2-Brie (78,637 sgRNAs targeting 19,674 genes and 1000 non-targeting sgRNAs)	4T1-GFP cells and mouse macrophage Raw264.7 cells *in vitro*, MOI = 0.5	Cells were subjected to phagocytosis assays GFP-labelled tumour cells cu-cultured with macrophages. Phagocytosis-positive macrophages were sorted by FACS and used for NGS analysis to identify receptor-ligand pairs that promote phagocytosis	Blocking Vitronectin (Vtn)- complement C1Q binding protein (C1qbp) interaction increases macrophage phagocytosis, inhibiting tumour progression *in vivo*	[128]
PD-L1 enhancer screening	Custom sgRNAs targeting PD-L1 enhancer region	SUM-159 cells *in vitro* arrayed screen	Western blotting for PD-L1 expression	NFE2L1/MAFG regulate PD-L1 transcription	[130]
*In vivo* screening identifies Mga as a driver of immune escape in TNBC	Mouse genome-wide CRISPR library	EMT6 cells implanted in BALB/c mice, TNBC syngeneic *in vivo* model, MOI = 0.25	Tumour growth, comparison of depleted genes between tumours in immunocompetent mice and *in vitro* cells	*Mga* was identified as a TNBC-specific gene that regulates immune evasion *in vivo* through regulation of MHCII expression	[131]
Tumour suppressor loss promotes inflammatory TME	Custom library of tumour suppressor and immunoregulatory sgRNAs	4T1 cells *in vivo*, MOI = 0.3	Cells injected into mammary fat pad, genomic DNA extracted from tumours and tissues containing metastases	Targeting LAG3 reduces metastasis in NF1-, TSC1- or TGF-β RII-inactivated cancers	(132)

## Identification of drivers of breast cancer

The use of CRISPR screens in breast cancer has significantly advanced our understanding of key drivers of tumourigenesis and metastasis. For example, an *in vivo* study using the human genome-wide GeCKOv2 library in TNBC SUM159PT cells identified both tumour suppressors and oncogenes [50]. In this study, 30 million cells were subcutaneously injected into severely immunodeficient NOD-SCID gamma (NSG) mice, after infection and puromycin selection. Following tumour formation and DNA sequencing, the analysis revealed many positively and negatively selected hits. *NF2*, *TSC1*, and *PTPN12* were positively selected, indicating a potential tumour suppressing function for these genes, while *MYC*, *HRAS*, and *PTPN11* were negatively selected, highlighting their oncogenic role. Functional validation implicated mTOR signalling in breast cancer progression, while pharmacological inhibition of mTORC1/2 and YAP reduced tumour growth through induction of apoptosis and promotion of macropinocytosis. This study highlights how CRISPR screens can be used *in vivo* to identify genes involved in breast cancer progression.

CRISPR screens have also been successfully employed to investigate the role of long non-coding RNAs (lncRNAs). Using a synergistic activation mediator (SAM) system, a study identified AK023948 as a positive regulator of AKT signalling [65]. AK023948 was shown to facilitate the interaction between DHX9 and p85, promoting AKT activity. The study further proposed that the upregulation of AK023948 and DHX9 may play a role in breast cancer progression, as DHX9 associates with poorer survival in breast cancer, highlighting lncRNAs as potential therapeutic targets.

Another study focusing on lncRNAs and how cryptic translation can drive breast cancer has also been carried out. They identified the lncRNA-encoded protein, GATA3-interacting cryptic protein (GT3-INCP) as a key driver of breast cancer associated with poor prognosis. They found GT3-INCP interacts with GATA3, promoting proliferation [66]. Their innovative approach used ribosome profiling to identify ribosome footprints and predict open reading frames. Cryptic open reading frame-targeting sgRNAs were then used in a library to identify lncRNA-encoded proteins associated with breast cancer growth.

Taking a different approach, another study used CRISPR-Cas13d to target 844 breast cancer-related lncRNAs [67]. The results showed that KILR sequesters RPA1 and that loss of KILR promotes growth through increased RPA1 and increased DNA replication speeds. The researchers also found that KILR overexpression promotes apoptosis. CRISPR-Cas13d RNA knockdown was used as CRISPR-Cas9 knockout is often ineffective for lncRNAs. Furthermore, the authors did not use CRISPRi as lncRNA transcription often initiates from enhancer elements in DNA [67]. CRISPR-Cas13d works through RNA-targeting to reduce gene expression, making it a useful tool for the study of lncRNAs [68].

Alternative splicing is another critical process in breast cancer that has been explored using CRISPR screens. One *in vivo* screen identified PHD finger protein 5 A (PHF5A) as a regulator of migration, survival, and breast cancer formation through SF3b spliceosome stability, suppressing apoptosis [51]. Another screen exploring the hippo pathway in breast cancer uncovered the transcriptional repressor protein Trichorhinophalangeal syndrome 1 (TRPS1) as a key repressor of Yes-associated protein (YAP)-dependent transactivation [69]. The study also found that high TRPS1 activity negatively correlates with YAP activity and results in reduced immune cell infiltration into tumours. Another study exploring RNA-binding proteins identified YTHDF2 as a driver of cell death in MYC-driven breast cancer given that loss of YTHDF2-dependent mRNA degradation induces apoptosis in TNBC [70].

Selection of an interesting and useful CRISPR library is the key starting point for CRISPR screening studies. One innovative study began with genome-wide association studies to assemble a library of sgRNAs targeting genes identified as associated with breast cancer [71]. Through sequential *in vitro* and *in vivo* screens using CRISPR knockout and CRISPR activation technologies, they identified many breast cancer risk genes that drive proliferation *in vitro* and *in vivo*.

A landmark study on circulating tumour cells (CTCs) used a genome-wide *in vivo* CRISPR activation screen to find genes that promote metastasis in mouse models [52]. CTCs were enriched from the blood of hormone receptor-positive metastatic breast cancer patients and used to generate cell lines. The cells were then injected into mice tail veins, and lungs were isolated to identify enriched sgRNAs. They found that overexpression of RPL15, a large ribosomal subunit component, strongly increased metastatic growth, demonstrating a link between ribosomal content and CTC metastatic ability.

A similar study also explored how CTCs form distal metastases [72]. They established a cell line from CTCs and used these in a loss-of-function CRISPR screen *in vivo*. Cells were injected into mammary fat pads of mice and allowed to grow. Primary tumours, CTCs, and metastases were then harvested for DNA extraction and NGS. The authors identified genetic dependencies for each step of metastasis, finally validating inhibitors that could block different stages. They identified PLK1 inhibition as a potentially useful strategy to block intravasation of CTCs.

Epithelial-mesenchymal transition (EMT) is a key step in carcinogenesis, and CRISPR screening has been applied to understand how this transition is regulated [73]. Using a genome-wide CRISPR knockout screen, one group identified regulators of EMT by sorting for cells with a mesenchymal phenotype through magnetic-activated cell sorting (MACS) and FACS for mesenchymal markers. They identified two distinct programs where PRC2 or KMT2D-COMPASS regulate the epithelial state, and loss of either of these induces a different EMT process.

A targeted CRISPR screen focusing on lipid metabolic genes identified *NSDHL* as a driver of metastasis in TNBC involved in TGFβ signalling and TGFβR2 degradation [53]. Mice were injected with MDA-MB-231LM2/Cas9/luciferase-GFP cells, and metastases were collected from the lung and subsequently sequenced. Results showed that inhibition of upstream metabolism of NSDHL with ketoconazole reduced cancer metastasis. The use of a targeted library in this study enabled greater depth and more reads per gene, allowing an *in vivo* study to be conducted into lipid metabolic genes to identify novel drivers of metastasis, highlighting the power of targeted screens in uncovering therapeutic targets within specific pathways.

CRISPRi has also been utilised for genome-wide and single-cell screens to explore transcriptional regulatory elements (TREs) up- and downstream of ERα in breast cancer [74]. Using a fluorescence-based reporter system to assess expression of *ESR1* and *CCND1* in MCF7 cells, TREs within the topologically associating domains (TADs) of *ESR1* and *CCND1* were targeted. The authors initially used proliferation-based CRISPRi screening to examine sgRNA depletion in the final pool. Next, they sorted the MCF7 cells by FACS based on *ESR1* and *CCND1* and identified sgRNAs with strong effects on both proliferation and gene expression. In a follow-up screen, they used a genome-wide CRISPRi library to identify ER binding sites (ERBS) in breast cancer using CRISPR droplet sequencing (CROP-seq) followed by single-cell RNA sequencing. This technology allows large amounts of information to be collected on gene expression patterns in a high-throughput manner [57]. CRISPRi screening can also be conducted on pseudogenes where one study found that approximately 70 pseudogenes have a role in breast cancer fitness [75]. In particular, MGAT4EP interacts with FOXA1, which enhances promoter binding of FOXA1 and increases expression of FOXM1, promoting oncogenesis.

Combinatorial CRISPR screening is another useful tool, particularly for examining synthetic lethal interactions; however, the total number of sgRNAs must be reduced to maintain sufficient depth due to cell culture constraints and the large number of gene pairs resulting from a small number of genes. One study used CROP-seq of MCF10A cells harbouring loss of *PTEN*, *PIK3CA*, or *MYC* oncogenic alterations for dual targeting of tumour suppressor genes to explore epistatic networks in breast cancer [76]. This approach leads to the identification of signalling networks rather than single driving genes, which can be invaluable in complex diseases such as breast cancer with multiple redundant pathways driving growth and other processes.

While 2D cell culture models are simpler to use, and *in vivo* models are better at capturing the native surroundings of tumours, 3D cell culture models can provide a balance. One study of miRNAs found miR-4787-3p to play a role in tumour-initiating cell growth [77]. The authors used assays measuring 2D and 3D growth of cells in order to identify miRNAs involved in tumour initiation. The top hits were later validated, highlighting the relevance of 3D models in CRISPR screening.

A recent study used CRISPR activation screening to identify factors promoting CTC survival and metastatic growth [49]. The group generated circulating tumour cell organoids from patients and identified neuregulin 1 (NRG1)-ERBB2 receptor tyrosine kinase 3 (ERBB3/HER3) signalling as a key survival pathway for cells through multiomics. Furthermore, they used CRISPRa screening to find that FGFR1 signalling can compensate for this in NRG1-deficient CTCs. Overall, they identified these pathways as important in CTCs and showed that combined treatment strongly inhibits growth of CTC-derived organoids *in vivo*.

Using a tumour suppressor-targeting sgRNA library in MCF10F cells, one group identified far upstream binding protein 1 (FUBP1) as a long tail driver of breast cancer [78]. Using a high MOI of 3, the authors ensured that cells would receive multiple sgRNAs, allowing them to explore combinations of sgRNAs and their effects on *in vivo* cell growth. In addition to well-established cancer drivers, they showed that loss of FUBP1 drives transformation and promotes tumour growth when PTEN is also lost.

CRISPR screens have also been used successfully to identify how particular genes act. Beclin 1 is a tumour suppressor, and it inhibits breast cancer growth. One study used a CRISPR screen to find genes which, when lost, abrogate the tumour suppressive function of Beclin 1 in order to understand how Beclin 1 inhibits cell proliferation [81]. They found that *CDH1* and *CTNNA1* were required for Beclin 1 to inhibit breast cancer cell growth by conducting a CRISPR screen in cells with and without Beclin 1 expression. This innovative strategy enabled the identification of genes involved in Beclin 1-mediated growth suppression, advancing our knowledge of this pathway in breast cancer. Specifically, they showed that Beclin 1 promotes E-cadherin localisation to the plasma membrane, restricting tumour growth and metastasis [81].

Another recent study employed an *in vivo* model for the identification of *Trp53* collaborating tumour suppressor genes. Using primary mammary cells obtained from *Trp53*^+/−^ BALB/c mice, researchers performed a genome-wide CRISPR screen *in vivo* and identified several tumour suppressor genes, such as *Axin1* and *Prkar1a*. Follow-up experiments using tumour organoids and direct intra-ductal genome editing showed that mutated *Axin1* or *Prkar1a*, combined with *Trp53* haplo-insufficiency, resulted in increased tumour growth [84].

CRISPR screens have been used to identify the contribution of aberrant enhancer activity to breast cancer growth. One such study identified 2500 super-enhancers in TNBC cells that were not active in normal breast tissue. Furthermore, by CRISPR screening these enhancers, they identified several tumour-specific dependencies. In particular, they showed that BMP and activin membrane-bound inhibitor (BAMBI) correlates with the super-enhancers and works as a key driver of TNBC growth [79].

Another study focused on super-enhancers that are transcribed in non-coding enhancer RNA (eRNA) in TNBC cell lines. Using an arrayed CRISPRi library, 30 super-enhancers were targeted *in vitro*, and transcriptomics revealed an intricate relationship between differentially expressed genes and eRNA. As a proof of concept, targeting a *PODXL*-associated super-enhancer resulted in downregulation of gene expression, decreased cell proliferation *in vitro*, and tumour growth *in vivo* [80].

Base editing is an innovative new technology where Cas9 nickase is fused with cytosine or adenine deaminase enzymes to introduce base changes in DNA [17]. Through a high-throughput CRISPR base editing screen, one group explored how mutations in DNA damage response genes could influence cellular fitness after DNA damage [82]. Through this screen, they found mutations in the Tudor domain of 53BP1 define a non-canonical surface for the binding of USP28. Additionally, they discovered variants in TRAIP ubiquitin ligase, defining a new domain that, when lost, caused resistance to topoisomerase I inhibition. Furthermore, they characterised CHK2 kinase loss of function mutations, highlighting the wealth of information that can be gained from such screenings.

PRIME editing has also been applied to breast cancer, where one study used it for functional characterisation of breast cancer-associated variants [83]. They assessed the impact of SNPs associated with breast cancer on cell fitness, identifying several as altering cell growth.

## Exploring sensitisers to chemotherapies

CRISPR screening is also being used to advance our understanding of how chemotherapies work in breast cancer, how resistance develops, and potential ways to overcome resistance. Paclitaxel is a chemotherapy drug used in TNBC with microtubule-stabilising properties that inhibit tumourigenesis by blocking the spindle checkpoint and inducing mitotic arrest [85]. However, resistance to taxols can occur through many different mechanisms, limiting the efficacy of this treatment. To address this, one group employed a kinome-wide CRISPR library to screen for genes implicated in paclitaxel resistance and identified PLK1, a key driver, which when silenced inhibits cancer growth *in vivo* and *in vitro* [45].

A subsequent investigation also utilised CRISPR screening to uncover sensitisers of paclitaxel in TNBC; however, a genome-wide approach using the GeCKOv2 library was taken [86]. Interestingly, this study consisted of both an *in vivo* and an *in vitro* screen, and candidate genes were selected from overlapping *in vitro* and *in vivo* sgRNA hits. The group found that many genes involved in stemness, including *ATP8B3*, *FOXR2*, *FRG2*, and *HIST1H4A*, enhance paclitaxel resistance when knocked out. Furthermore, upregulation of FRG2 through CRISPRa sensitises tumours to paclitaxel treatment and reduces their metastatic potential.

Another targeted library of 2240 ‘druggable’ genes was used in a loss-of-function screen to uncover TNBC vulnerabilities [87]. This library targets kinases, FDA-approved drug targets, DDR genes, chromatin modifiers, and several other genes involved in cancer, and was selected to enable druggable hits to be identified, increasing translational potential. In this study, cells were treated with 5-fluorouracil (5-FU) or everolimus for 2 to 4 weeks, showing that epithelial-like cells are more vulnerable to EGFR-RAS-MAPK loss, while mesenchymal cells are more vulnerable to G2-M cell cycle regulator loss. Furthermore, EGFR and fatty acid synthase (FASN) were highlighted as critical druggable targets for sensitisation of cells to these therapies.

An arrayed CRISPR screen on deubiquitinating enzymes (DUBs) involved in YM155-induced cytotoxicity response was carried out in MCF7 cells infected with sgRNAs. Subsequently, proliferation was measured during YM155 treatment to find DUBs conveying resistance to YM155 [88]. The focused nature of the DUB-based screen meant an arrayed screen would be more cost-effective and simpler to carry out. Interestingly, the screen revealed USP32 as a destabiliser of SLC35F2, reducing uptake of the chemotherapeutic agent.

## Overcoming radiotherapy resistance

Radiotherapy is a cornerstone treatment for brain metastases; however, its efficacy is limited by the poor therapeutic index and damage to surrounding cells. To overcome this, brain metastases could be sensitised to radiotherapy by impairing their DNA damage repair abilities, therefore improving treatment efficacy at lower doses. Using a genome-wide screen, a study showed that LRRC31 can inhibit DNA-PKcs recruitment and disrupt MSH2-ATR [89]. The CRISPR screen used a lethal dose of radiation to select for cells with sgRNAs that enable survival after exposure. The authors then inferred that the upregulation of these genes could confer radiosensitivity. They experimentally determined this for LRRC31 and found the mechanism that involved an interaction with Ku70/Ku80 and ATR. The authors also showed that delivery of LRRC31 to mice via nanoparticles improves their survival following radiotherapy.

A later study used whole-genome CRISPR screens in MCF10A cells to explore radiation response in breast cancer [90]. Interestingly, the authors selected epithelial rather than breast cancer cells due to their reduced heterogeneity and genetic alterations, allowing the exploration of more responses to radiotherapy. They showed that BCL2L1 inhibition combined with radiotherapy reduces tumour growth more effectively than radiotherapy alone and propose BCL2L1 inhibition as a potential combination anti-cancer therapy.

## Identification of drivers of endocrine therapy resistance

Endocrine therapies form the foundation of treatment for ER + breast cancers, which constitute around 70% of all breast cancers [91]. However, both primary and acquired resistance limit therapeutic efficacy [92]. Therefore, the identification of mechanisms causing endocrine therapy resistance in breast cancer is a crucial step to overcoming this obstacle. One group carried out a pooled loss-of-function CRISPR screen to find drivers of resistance in ER + cells [46]. By culturing ER + cells with and without oestrogen, they identified key ER + breast cancer essential genes and genes regulated by oestrogen. Once an interesting hit, *CSK*, was identified, the authors performed a second screen to find genes synthetically lethal with loss of *CSK*. Ultimately, they identified a feedback loop that limits the efficacy of endocrine therapies where CSK is induced by oestrogen, and in the absence of oestrogen, cells have low levels of CSK, which activates protein-activated kinase 2 (PAK2), driving breast cancer growth. A later study used a similar approach to identify PAICS inhibition as a sensitiser to tamoxifen [93]. Furthermore, another study used genome-wide CRISPR screening to find Tafazzin as a mediator of tamoxifen resistance in breast cancer [94].

Taking a different approach, one group explored chromatin structure to identify whether CTCF-binding elements (CBEs) are essential for ERα-driven cell proliferation using a library targeting CBEs close to ERα-binding sites [48]. In this study, the authors compared the ER + MCF7 with the TNBC MDA-MB-231 cell line to look for ER + BC-specific sgRNAs, finding that one CBE had a role in PREX1 activation, which is associated with favourable outcomes following endocrine therapy.

Targeted screens are helpful in identifying genes in specific categories that are essential for particular processes. Using this approach, one group showed that *ARID1A* loss conveys resistance to fulvestrant [95]. Using an epigenome-associated sgRNA library, they showed that *ARID1A* mutations lead to resistance to ER degraders as loss of *ARID1A* plays a key role in loss of luminal identity in ER + breast cancer cells.

Although anti-oestrogen drugs are the most common form of endocrine therapy, in a subset of advanced or metastatic ER + patients, administration of oestrogens can elicit anti-cancer effects [96, 97]. To understand how this occurs, one study used a genome-wide CRISPR screen identifying that oestrogen treatment increases replication-dependent DNA damage markers and apoptosis, and that PARP inhibition can be used synergistically with oestrogens to suppress growth [98]. Using HCC-1428 cells (dependent on oestrogen for growth) and long-term oestrogen-deprived (LTED) derivative cells, the authors found essential genes in the presence and absence of oestrogen, pinpointing pathways and genes involved in oestrogen-dependent growth, many of which are involved in DNA repair and cell cycle progression. Later experiments showed that oestrogen induces DNA damage response and transcriptional stress.

Another important study used genome-wide CRISPR screening in MCF7 cells to explore which genes were required for the response to tamoxifen and fulvestrant [99]. Interestingly, they found more than half of the genes required for fulvestrant to exert its anti-cancer effects were also required by tamoxifen. One of the BAF ATP-dependent chromatin remodelling complex components, AT-Rich Interaction Domain 1 A (ARID1A), was among the most significant hits. The authors showed that *ARID1A* mutations occur in treatment-resistant breast cancer and may provide a rational treatment strategy.

## Exploring drivers of HER2 therapy resistance

Anti-HER2 therapies, such as trastuzumab, are a key part of the treatment of HER2 + breast cancer; however, resistance to these therapies remains a significant clinical challenge. One study aimed to identify mediators of anti-HER2 therapy resistance by conducting a genome-wide CRISPR screen *in vivo* and *in vitro* [100]. By treating cells with trastuzumab, the authors identified genes involved in resistance to HER2 therapies. Notably, they discovered that upregulation of fibroblast growth factor receptor 4 (FGFR4) confers anti-HER2 therapy resistance through regulation of ferroptosis. Mechanistically, they found that FGFR4 phosphorylates GSK-3β and activates β-catenin/TCF4 signalling, promoting resistance to anti-HER2 therapies. They also suggest that FGFR4 inhibition could potentially restore sensitivity to anti-HER2 therapies.

Another investigation explored regulators of breast cancer metastasis, focusing on autophagy-related genes [54]. The authors used N148 cells infected with a custom library targeting 171 genes in order to focus the screen on genes involved solely in one pathway. The cells were orthotopically injected into mice and allowed to form tumours. Given that these cells were non-metastatic, the authors reasoned that lung metastases would include sgRNAs targeting regulators of this process. Sequencing revealed that p47 is a suppressor of lung metastasis, where it influences NF-κB signalling and autophagy, revealing another pathway that could be exploited to improve treatment of HER2 + breast cancer.

## Finding therapies for BRCA-mutant cancers

In cells deficient in homologous recombination, such as those with *BRCA1/2* mutations, PARP enzyme inhibition causes cell death. This is particularly relevant in BRCA-mutant breast cancers, where PARP inhibitor (PARPi) therapies are a useful approach through synthetic lethality; however, resistance is a major obstacle [101]. To identify factors contributing to PARPi resistance in breast cancer cells, one study used loss-of-function CRISPR screening [102]. In three separate screens, cells were treated with different PARPi therapies following infection with different libraries of sgRNAs. The authors found that loss of CTC1-STN1-TEN1 (CST) complex components induced PARPi resistance in BRCA1-deficient cells, uncovering potential therapeutic implications. Another study took a similar approach to look for genes synthetically lethal with *BRCA1* deficiency and PARP inhibition [103]. In a genome-wide screen, the authors identified C20orf196 and FAM35A inactivation as strong promoters of PARPi resistance. Another study used genome-wide and high-density CRISPR-Cas9 tag-mutate-enrich mutagenesis screening to identify mutations that confer resistance to PARPi. Following treatment with talazoparib, cell populations containing *Parp1* mutations were identified, showing increased PARPi resistance [104].

To expand the scope of treatments for BRCA-mutant cancers, one group used CRISPR screening to identify synthetic lethal interactors of *BRCA1* in addition to PARP [105]. They used a custom library composed of 12,500 sgRNAs targeting 334 epigenetic regulators and 657 genes for which FDA-approved drugs already exist. The screen revealed *MEPCE*, a methylphosphate capping enzyme, as a synthetic lethal interactor of *BRCA1*. Loss of *MEPCE* causes dysregulated RNA polymerase II (RNAPII) promoter-proximal pausing, R-loop accumulation, and replication stress, all of which increase transcription-replication machinery collisions, causing cell death.

Another important study explored additional therapeutic targets in homologous recombination-deficient cells and identified the PAR-binding chromatin remodeller, ALC1/CHD1L, as a crucial protein for PARPi toxicity [106]. A library targeting chromatin regulators was used in order to identify sensitisers to olaparib in BRCA-mutant breast cancer cell lines. The authors showed that *ALC1* loss increases genome instability and therefore reliance on BRCA-dependent mechanisms of homologous recombination. Through this mechanism, *ALC1* loss promotes sensitivity to PARP inhibition in HR-deficient cells, suggesting a potential therapeutic approach to overcome resistance to these drugs.

## Understanding CDK4/6 inhibitor resistance mechanisms

CDK4/6 inhibitors (CDK4/6i), such as palbociclib, have proven useful in the treatment of advanced ER + breast cancer; however, resistance to these therapies poses a significant challenge. In a genome-wide CRISPR screen, one group examined how palbociclib resistance develops in MCF7 cells [107]. The authors found that coagulation factor IX (F9) loss prevents cell cycle arrest and senescence in breast cancer cells treated with palbociclib. They propose that F9 could be used as a potential biomarker to predict CDK4/6i response in cancer therapy. Another CRISPR-Cas9 screen used a CRISPR knockout library targeting transcription factors and epigenetic molecules in MCF7 cells [108]. This alternative approach led to the identification of *GATAD1* as a synthetic lethal target with CDK4/6 inhibition in breast cancer through the induction of cell cycle arrest. A later study also used genome-wide CRISPR screening and identified SEMA3F as a potential regulator of resistance to CDK4/6 inhibitors [109].

More recently, another genome-wide CRISPR screen identified PRMT5 as a vulnerability in ER + BC lacking *RB1* [110]. *RB1* knockout cells were used due to the prevalence of *RB1* mutations in breast cancer conveying resistance to CDK4/6 inhibitors. The study found that PRMT5 inhibition blocks the G1/S transition in breast cancer cells, arresting cell growth. This study suggests that PRMT5 inhibition could be a potential therapy in overcoming CDK4/6i resistance in advanced ER + breast cancer.

Using CRISPR screens *in vivo* can greatly enhance their translational ability by better mimicking the tumour microenvironment of breast cancers. A genome-wide *in vivo* CRISPR screen in TNBC for sensitisers of palbociclib was performed and showed that TGFβ3 inhibition can sensitise tumours to CDK4/6 inhibition [111]. The authors used the SUM159PT cell line due to its *Rb* positivity, making it susceptible to CDK4/6 inhibitor treatment, and the presence of *PI3KCA* and *TP53* mutations which are highly prevalent in TNBC [112, 113]. They used a genome-wide CRISPR knockout library (GeCKOv2) to transduce cells *in vitro* which were then subcutaneously injected into NSG mice. The mice were treated with palbociclib, and tumours were removed after 30 days. After sequencing, computational analysis was used to further narrow down the hits to eight genes. These eight genes were then individually knocked out in SUM159PT cells and then orthotopically transplanted into mammary fat pads of NSG mice, and the results of the initial screen were validated. Finally, the authors showed that TGFβ3 inhibition is synergistic with palbociclib in a p21-dependent manner. Palbociclib depletes p21 from cells, whereas TGFβ3 restores levels of p21, allowing continued inhibition of CDK/cyclin complexes that often disappears following palbociclib treatment.

A recent genome-wide CRISPRa screen identified METTL14 as a driver of CDK4/6i resistance in ER + BC. Mechanistically, researchers discovered that CDK4/6 inhibitors reduce the ubiquitination and degradation of METTL14. Higher protein levels of METTL14 promote the stabilisation of E2F mRNA via increased METTL14-mediated m6A modification and IGF2BP2-mediated mRNA stability, resulting in increased E2F translation and cell cycle progression. Treatment of CDK4/6i resistant cell lines and PDX models with a novel METTL14 inhibitor, WKYMVM, resulted in the re-sensitisation to CDK4/6i therapy [114].

## Exploring novel targeted therapies and combination treatments

In addition to more traditional therapies, CRISPR screens have also been conducted to identify sensitisers of cells to novel therapies. Threonine tyrosine kinase (TTK) is a regulator of the mitotic spindle assembly checkpoint, and its inhibition has been explored as a therapy in breast cancer [115]. Using a CRISPR-Cas9 knockout screen, one group found genes involved in conferring resistance to the TTK inhibitor, CFI-402257 [116]. Anaphase-promoting complex/cyclosome (APC/C) components were found to be significantly enriched as resistance factors and potential combination therapeutic targets.

Another innovative novel therapy involves antibody–drug conjugates (ADCs), which enhance the precision of chemotherapies by targeting cancer cells directly. A CRISPR screen identified C18ORF8/RMC1 as a novel regulator of ADC toxicity, working through regulation of endolysosomal processing [117]. They showed that sialic acid depletion can enhance the delivery of ADCs and improve killing of cancer cells, offering a potential strategy for improving the efficacy of these therapies.

In non-small cell lung cancer, EGFR inhibition is an effective strategy; however, in TNBC, it proves ineffective, despite similar levels of EGFR activation. To investigate this, a CRISPR knockout screen was conducted, revealing that the elongator (ELP) complex promotes insensitivity to EGFR inhibitors in TNBC [118]. The authors used a genome-wide library and treated cells with the EGFR inhibitor, erlotinib, to find depleted sgRNAs. They identified ELP proteins, which, when depleted, induce apoptosis when EGFR is inhibited in TNBC. Another study also explored sensitisers to EGFR inhibitors and identified CDK12/13 as targets that could be used in combination with EGFR inhibitors [119].

Combination therapies have also been explored to enhance the efficacy of existing drugs that fail to show efficacy in breast cancer. For instance, the Aurora‐A inhibitor, alisertib (MLN8237), proved ineffective in clinical trials [120]; therefore, a study was carried out to find kinases synthetically lethal with MLN8237 [121]. Using a targeted kinase sgRNA library allowed the researchers to identify targetable proteins with more depth, and it uncovered histone H3 associated protein kinase (Haspin) as a promising target. Through this approach, researchers showed the combination worked synergistically to reduce breast cancer growth *in vitro* and *in vivo*. Mechanistically, they showed that these inhibitors abolish Aurora-B and mitotic centromere-associated kinesin (MCAK) recruitment to centromeres, causing mitotic catastrophe.

PI3Kα inhibitors are another class of drug that may have therapeutic potential in breast cancer. Through a genome-wide CRISPR knockout screen, a group found that loss of negative mTORC1 regulators confers resistance to PI3Kα inhibition, suggesting that mTOR inhibitors could serve as potential combination partners to overcome resistance [47]. The authors used *PIK3CA*-mutated ER + MCF7 and T47D PI3Kα-sensitive cells, and they used alpelisib or taselisib to inhibit PI3Kα, uncovering several genes responsible for PI3Kα inhibitor sensitivity [47]. MEK inhibitors have also been explored for breast cancer treatment; however, due to their poor efficacy, one group sought to identify sensitisers through a genome-wide CRISPR screen, showing that PSMG2 inhibition increases the efficacy of the MEK inhibitor AZD6244 [122]. A similar approach has also been used that identified integrin-linked kinase (ILK) as a sensitiser to SRC inhibition [123]. These studies highlight the potential of CRISPR screens to uncover new synergistic therapeutic strategies for breast cancer.

Another candidate therapeutic for breast cancer is BET bromodomain inhibitors that elicit anti-proliferative and selective transcriptional responses in TNBC. One study aimed to find additional synthetic lethal interactions with BET bromodomain inhibitors [124]. In particular, they found that CDK4 and BRD2 were the top synthetic lethal hits, whereas BRD7 loss can promote resistance to BET bromodomain inhibitors.

Another study employed a combinatorial CRISPR library in order to identify gene pairs that could be used as a combinational therapy in TNBC. The simultaneous inhibition of the tyrosine kinases KDM4 and FYN potentiated the therapeutic effects of the TKI inhibitors imatinib, gefitinib, and NVP-ADW742. TKIs promote the expression of KDM4, which then promotes FYN transcription by demethylating its promoter region, ultimately promoting TKI therapy resistance [125].

## Improving breast cancer immunotherapies

The tumour microenvironment plays a pivotal role in the initiation, development, and progression of breast cancer. Several key processes are dependent on the interactions between cancers and the tumour microenvironment, including angiogenesis, metastasis, immune evasion, and response to therapy. Advances in CRISPR screening have allowed the systematic identification of genes involved in these interactions, with research focusing heavily on the immune compartment of the microenvironment. One landmark study explored CD8 + T cells in breast cancer by carrying out a genome-wide *in vivo* CRISPR screen [55]. The authors found that RNA helicase DHX37 knockout enhances anti-tumour immunity in T cells, showing that DHX37 suppresses cytokine production and T cell activation and that DHX37 interacts with PDCD11 influencing NF-κB activity. This *in vivo* model allowed researchers to perform autologous translation of the T cells and identify genes involved in tumour infiltration by extracting tumour infiltrating lymphocytes and exploring enriched sgRNAs.

Another CRISPR-Cas9-based screen in T cells identified p38 kinase as a regulator of T cell expansion, differentiation, oxidative stress, and genomic stress [126]. With the aim of identifying enhancers of T cell anti-cancer activity, the authors first identified four readouts for high-throughput single-cell assays in order to identify favourable phenotypes in T cells. Unlike most other studies, they used an arrayed CRISPR knockout screen in primary mouse T cells to target 25 kinases related to T cell receptor (TCR) stimulation. One protein, p38, was validated as a regulator of phenotypes in T cells, and its inhibition enhanced T cell activity, suggesting that inhibitors of p38 may have therapeutic potential. Using an arrayed study allowed the use of four different readout phenotypes in one experiment measured by flow cytometry rather than sequencing. The small number of kinases made this a feasible and effective approach.

Macrophages are another key immune cell type in breast cancer and may serve as promising, yet underexplored immunotherapy targets. One study utilised pooled *in vivo* CRISPR knockout screening in syngeneic TNBC models to show that Cop1 deletion in breast cancer cells reduces macrophage infiltration into tumours, improving response to immune checkpoint blockade [56]. The authors compared the growth of CRISPR library-treated cells in immunocompetent and nude mice to find drivers of immune evasion. A secondary screen was also conducted with 79 genes from the primary screen and 8 sgRNAs per gene to increase robustness. Cop1 was validated and proposed as a target for improving immunotherapy in TNBC. Another study used a CRISPR library of disease-related immune genes to identify galectin-2 as a regulator of immune escape and M2 polarisation [127]. The use of a targeted library increased the confidence in the results, and mechanistic experiments revealed that tumour galectin-2 induced tumour-associated macrophage infiltration, M2 polarisation, and colony stimulating factor 1 (CSF1)/CSF1 receptor (CSF1R) signalling in macrophages promoting their proliferation.

Another study used a CRISPR screening approach to identify genes related to macrophage phagocytosis [128]. This genome-wide CRISPR screen showed that blocking the vitronectin (Vtn)-complement C1Q binding protein (C1qbp) interaction could increase macrophage phagocytosis, inhibiting tumour progression *in vivo*. They took an interesting approach of labelling tumour cells with GFP and subsequently exposing them to macrophages before sorting macrophages using FACS to find more or less phagocytic cells.

Additionally, CRISPR screens have been used to identify genes that modulate response to immune checkpoint inhibitors. One such study used an *in vitro* tumour immune cell co-culture system to assess genes involved in immune checkpoint blockade in TNBC [129]. This study used primary lymphocytes from healthy volunteers and MDA-MB-231 cells infected with a genome-wide sgRNA library. The cells were co-cultured with and without nivolumab to find genes involved in vulnerability to anti-PD-1 therapies. Although an *in vitro* system was used, the results were confirmed *in vivo*, validating the co-culture system as a method of conducting CRISPR screens. Ultimately, they found that *NEDD8* loss increases sensitivity to nivolumab (anti-PD-1) using *in vivo* models of breast cancer.

Focusing on PD-L1 expression, another study used saturated CRISPR screening of the enhancer region to find loci essential for expression of PD-L1 [130]. The authors designed sgRNAs to target different regions, identifying locus 22 as essential for PD-L1 expression. They then found that NFE2L1/MAFG-based transcriptional regulation was important in PD-L1 expression through BRD4 binding. The authors finally showed that silencing of these transcriptional regulators or modification of locus 22 resulted in a reduction in T cell-mediated killing, identifying novel regulators of PD-L1 expression.

*In vivo* CRISPR screens can generate valuable information when studying immune regulation. A study employed a genome-wide CRISPR screen in various types of cancer, including TNBC using *in vivo* models, and identified Mga as a regulator of immune escape in TNBC. Depletion of *Mga* resulted in inhibition of tumour growth, but only in immunocompetent mice, and not *in vitro*. Transcriptome analysis and scRNAseq revealed that Mga affects T cell-mediated anti-tumour immunity, through regulation of MHCII expression [131].

Another *in vivo* CRISPR screen used 4T1 cells injected into mice for a pooled screening [132]. A library was designed to include tumour suppressor genes and genes associated with changes in tumour immune microenvironment activity. The cells were injected into mice, and genomic DNA was isolated from metastases to find genes associated with metastatic colonisation. Both nude mice and immunocompetent mice were used in order to find how the immune system contributes to these effects. They showed that NF1, TSC1, and TGF-β RII are important tumour suppressors that regulate immune composition, and their loss enhances IL6-JAK3-STAT3/6 inflammatory pathways, increasing LAG3 + CD8 and CD4 T cells. They further showed that LAG3 and PD-L1 targeting therapies inhibit metastasis in NF1-, TSC1-, or TGF-β RII-deficient tumours.

**Fig. 3:**
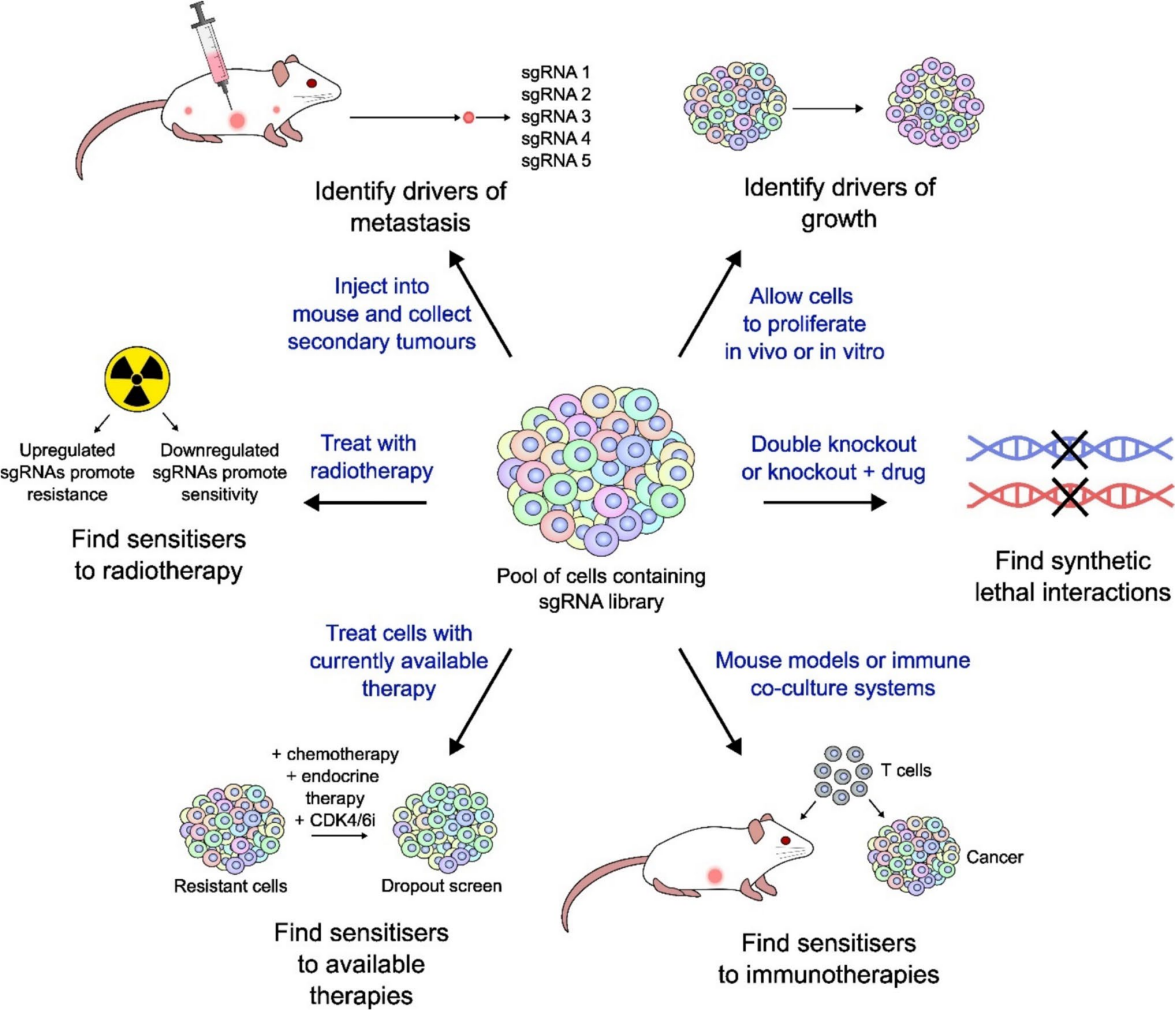
Applications of CRISPR-Cas9 screening experiments. Schematic representation of the different experimental procedures commonly used in breast cancer research. The figure shows how pooled CRISPR screens can undergo selection in different systems to identify drivers of oncogenic processes, such as metastasis, growth, and sensitivity to therapies, and in order to find synthetic lethal gene or drug interactions

Together, these studies illustrate the utility of CRISPR-based screening in understanding the complex interactions of cancer cells with surrounding immune cells. Through the identification of new genes involved in these interactions, screening studies offer new approaches for therapeutic development in modulating the response to immunotherapies, improving cancer treatment and patient outcomes (Fig. 3).

## Challenges and limitations of CRISPR screenings

CRISPR-based screening has revolutionised the study of gene function and identification of synthetically lethal interactions in breast cancer; however, significant challenges and limitations remain. Major challenges include potential off-target effects, sgRNA design complexities, the inter- and intra-tumour heterogeneity of breast cancers, selection of appropriate and relevant models, and the need for rigorous validation of identified hits [133].

Off-target effects are a major issue with sgRNAs and can often lead to false-positive results. Recent advances in computational tools and the availability of optimised sgRNA libraries have reduced the issues associated with this challenge; however, robust validation of results remains essential [134]. This can be resource-intensive when large datasets are generated from high-throughput screens; however, secondary screens can address this limitation. By re-evaluating top hits from primary screens using independent methods, such as alternative sgRNAs, siRNAs, or pharmacological inhibitors, secondary screens can identify the top candidate genes with more confidence.

The selection of the most appropriate and relevant model is another key consideration in designing CRISPR-Cas9 screening experiments. While *in vitro* studies offer simplicity and improved scalability, they fail to fully replicate the complexity of the tumour microenvironment, compared to *in vivo* studies. However, while *in vivo* studies better capture the interactions in the tumour microenvironment, they present other limitations, such as a reduced maximum library size and greater intrinsic variability [135]. Patient-derived cells provide improved clinical relevance compared to immortalised cell lines; however, the large quantity of cells needed to maintain sufficient library coverage makes the use of many primary cell lines infeasible. Immortalised cells, however, present their own challenges as they frequently fail to capture the biology of primary tumours.

Another key consideration is to select the screening system most appropriate for the question being addressed. While CRISPR knockout screens are most common, they provide different insights compared to CRISPR activation or interference screens. Furthermore, the choice of readout plays a key role in studies and can range from simpler readouts such as proliferation or invasion to more complex readouts such as reporter-based assays or *in vivo* models of metastasis [11]. Therefore, tailoring readouts to each specific research question is crucial to maximising the overall impact of CRISPR screens.

## Future directions

Recent advances in CRISPR technology have had a profound impact on the relevance of screenings and their translational impact. For instance, the use of 3D culture systems in CRISPR screening can more accurately recapitulate *in vivo* conditions compared to 2D culture models [136]. Furthermore, the development of more advanced 3D co-culture systems accounting for different cell types of the tumour microenvironment and conditions of *in vivo* tumours will be invaluable in future CRISPR screening research. Patient-derived cells, such as circulating tumour cells, are another useful tool that will be powerful in studying the behaviour of human breast cancer cells [49]. *In vivo* models are also becoming more complex. Behaviours like metastasis can now be measured *in vivo* by injecting cells into mice orthotopically, subcutaneously, or through the tail vein and then sequencing DNA from the lungs or liver or other organs where metastases form [52]. This insightful approach allows researchers to find which genes are involved in the dissemination of cancer cells and any specific organotropism associated with different genes.

Emerging CRISPR technologies, including base and prime editing, also provide new directions. These technologies enable the introduction of specific, targeted insertions, deletions, and substitutions without causing DNA double-strand breaks and could be used to study cancer mutations or gene variants and their association with therapy resistance [137]. In addition to these, CRISPR-based epigenetic editing allows the study of epigenetic changes in cancer cells that could lead to the identification of epigenetic therapeutic targets [137]. CRISPR screening also presents many new opportunities for immunotherapy research. As immunotherapies continue to evolve, new fields are expanding, and CRISPR-based screenings can provide novel insights into these areas. Studies have already begun to explore sensitisers to immunotherapies and how to target T cells in breast cancer through CRISPR screens [55, 126]; however, exploring other tumour microenvironment cell types could offer further insight into immunotherapies in breast cancer.

CRISPR-based screens are not limited to protein-coding genes and have been used successfully to dissect the function of enhancers, which are regulatory elements in DNA that alter transcription of distal genes. Through CRISPR-based enhancer screening, sgRNAs are designed to target Cas enzymes to these regulatory regions to identify the effects of these regulatory elements on a particular phenotype or the expression of a particular gene [138]. One such study generated an sgRNA library to target p53-bound enhancers to find regulators of oncogene-induced senescence [138]. In a second screen, the authors also identified ERα-bound enhancers required for proliferation through a dropout screen. These screens identified regions of DNA that are essential for proteins to mediate their effects, highlighting the versatility of CRISPR screens. Another screen used CRISPRi to find genome-wide targets of non-coding enhancer RNA (eRNA)-producing super-enhancers by inhibiting the top 30 super-enhancers in order to find the genome-wide consequences, showing important roles of the enhancer elements in driving oncogenic gene expression programs [80].

The use of combinational CRISPR screens has been in development over the latest years, where two groups of targets can be used to create double-knockout (DKO) libraries. DKO CRISPR screens that target gene pairs can reveal synthetic lethal interactions between genes and provide new therapeutic approaches in cancer [139, 140]. These tools could be used to uncover genetic interactions between oncogenic pathways, such as the KRAS pathway, that could be used as a combinational therapy in the clinic [141]. The discovery of synthetic lethal interactions between paralog genes or highly inactivated tumour suppressors and targetable genes is a promising field in cancer research, as it offers specialised solutions in tumours bearing specific alterations [139].

The integration of state-of-the-art multiomics technologies with CRISPR screening vastly increases the data generated from CRISPR studies. Typical readouts from CRISPR screens are proliferation, migration, or fluorescence-based; however, many CRISPR screens are now integrated with single-cell RNA sequencing. This technology enables researchers to see the broader impact of different perturbations of the transcriptome of the cells, often leading to highly impactful discoveries, especially when *in vivo* studies are used [142]. Spatial transcriptomics and proteomics further enhance these studies by mapping gene expression and proteomic profiles within the tumour microenvironment. Future studies will likely build on these advancements by integrating more sophisticated multiomics approaches to further uncover gene functions, tumour microenvironment interactions, immune responses, and potential targets in breast cancer [143].

## Conclusions

Overall, CRISPR screening is an extremely powerful tool for understanding gene functions in breast cancer. A vast number of studies have been conducted into diverse areas of breast cancer biology, exploring genes that drive tumour growth, invasion, and metastasis, as well as identifying mediators of resistance to chemotherapy, radiotherapy, endocrine therapies, and novel treatments such as targeted drugs and immunotherapies. Recent advances in CRISPR technology such as single-cell sequencing, double-knockout screens, and the availability of Cas variants designed to fit a particular experiment have significantly expanded the scope and depth of these studies. As the field advances, CRISPR-based screening approaches will undoubtedly play an increasingly crucial role in the identification of novel therapeutic targets and the development of new treatment strategies for breast cancer.

## Data Availability

No datasets were generated or analysed during the current study.
